# Charting Endocrine Progenitors Across Species and Organs

**DOI:** 10.1002/advs.202522011

**Published:** 2026-08-30

**Authors:** Changying Jing, Michael Sterr, Özüm Sehnaz Caliskan, Lama Saber, Nicole Katarina Rogers, Samuel Ogden, Kei Kozawa, Christos Karampelias, Jessica Jaki, Aimée Bastidas‐Ponce, Falk Janos Farkas, Oliver Czarnecki, Osvaldo Rodrigues Pereira, Perla Cota, Valerie Vandenbempt, Diego Balboa, Anika Böttcher, Natalie Krahmer, Mohammad Lotfollahi, Julie B. Sneddon, Heiko Lickert, Mostafa Bakhti

**Affiliations:** ^1^ Institute of Diabetes and Regeneration Research Helmholtz Munich Neuherberg Germany; ^2^ German Center for Diabetes Research (DZD) Neuherberg Germany; ^3^ Munich Medical Research School (MMRS) Ludwig Maximilian University (LMU) Munich Germany; ^4^ Institute of Diabetes and Obesity Helmholtz Munich Neuherberg Germany; ^5^ School of Medicine Technical University of Munich (TUM) Munich Germany; ^6^ Wellcome Sanger Institute Wellcome Genome Campus, Hinxton Cambridge UK; ^7^ Instituto De Biotecnología Universidad Autónoma Nacional De México Mexico; ^8^ Stem Cells and Metabolism Research Program Faculty of Medicine University of Helsinki Helsinki Finland; ^9^ Cambridge Stem Cell Institute University of Cambridge Cambridge UK; ^10^ Department of Cell and Tissue Biology University of California San Francisco California USA; ^11^ Department of Anatomy University of California San Francisco California USA; ^12^ Diabetes Center University of California San Francisco California USA; ^13^ Eli and Edythe Broad Center of Regeneration Medicine and Stem Cell Research University of California San Francisco California USA

**Keywords:** cross‐species, endocrinogenesis, intestine, pancreas, progenitors

## Abstract

Hormone‐producing cells in the pancreas and intestine regulate whole‐body metabolism and are dysregulated in obesity and diabetes. A deeper understanding of endocrine lineage formation (endocrinogenesis) in these organs is essential to elucidate the healthy state and disease trajectories. Here, we performed cross‐species (mouse and human) and cross‐system (in vivo and in vitro) comparisons of pancreatic endocrine progenitors (EPs), complemented by cross‐organ (pancreas and intestine) analyses, using single‐cell transcriptomics and epigenomics, together with mouse bulk proteomics. We uncovered conserved and distinct gene regulatory networks (GRNs) and cell‐cell communication patterns during lineage allocation across species, systems, and organs. Additionally, we identified diabetes‐associated genes restricted to embryonic pancreatic EPs but largely absent in the adult pancreas, suggesting that dysregulation during development may predispose to diabetes. Furthermore, our multi‐source EP profiling resolved the transcriptional programs underlying the aberrant, in vitro‐enriched enterochromaffin cell formation during differentiation toward human islet cells, suggesting that these cells may not correspond to a naturally occurring cell type present in vivo development. Finally, we revealed conserved, dynamic shifts in the cell cycle and cytoskeletal organization, alongside divergent mRNA translation, during endocrinogenesis. Collectively, this multi‐source EP profiling establishes a resource mapping the molecular landscape of endocrinogenesis across two major metabolic‐endocrine organs.

## Graphical abstract

Endocrine progenitors give rise to the hormone‐producing cells of the pancreas and intestine. Using single‐cell multiomics and proteomics, this study compares these progenitors across species, systems, and organs, mapping the conserved and species‐specific gene regulatory networks that guide their formation. It further shows that stem cell–derived enterochromaffin cells likely lack an in vivo counterpart.

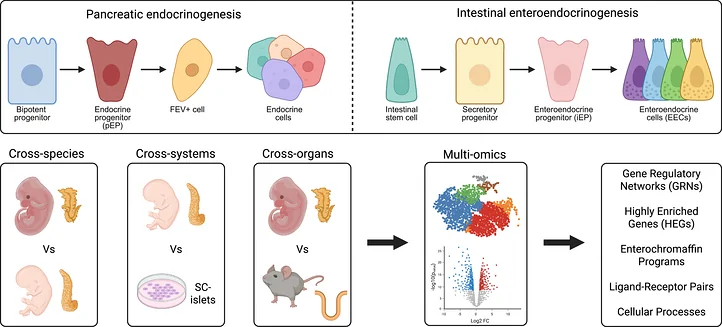

## 1 Introduction

In mammals, systemic metabolism is tightly regulated by coordinated humoral cross‐talk among organs to maintain energy homeostasis. Hormones from endocrine organs, particularly the pancreas and intestine, play a central role in regulating food intake and energy metabolism. Pancreatic hormones such as insulin, glucagon, and somatostatin maintain glycemic control, whereas gut‐derived hormones including glucagon‐like peptide‐1 (GLP‐1), glucose‐dependent insulinotropic polypeptide (GIP) and peptide YY (PYY), regulate insulin secretion as well as appetite and food intake [1, 2]. Despite the discovery of life‐saving insulin therapy for type 1 diabetes (T1D) and the emergence of potent anti‐obesity drugs, such as semaglutide and tirzepatide (a dual GIP/GLP‐1 receptor agonist) [3], these hormone‐based therapies largely manage symptoms rather than target underlying pathomechanisms of the disease. This underscores the need for strategies targeting the root cause of the pathology. For example, β‐cell regeneration and cell replacement could restore endogenous insulin secretion, reduce reliance on glucose monitoring and insulin injections and stably maintain blood glucose in a normal range [4]. Likewise, proof‐of‐concept gene therapies, such as engineered epidermal cells programmed to secrete GLP‐1, have normalized metabolic control in diet‐induced obesity and diabetes models [5]. Developing such causal therapies requires a deeper understanding of (entero)endocrine cell genesis (endocrinogenesis) in the pancreas and intestine.

Pancreatic endocrinogenesis is a transient process occurring during embryonic and fetal development, when bipotent progenitors within the pancreatic epithelium differentiate into EPs, marked by the expression of the transcription factor (TF) neurogenin‐3 (NEUROG3 or NGN3). Pancreatic EPs (pEPs) subsequently give rise to hormone‐producing cell types, including α‐, β‐, δ‐, ε‐, and pancreatic polypeptide (PP) cells [6, 7]. Despite their central role, the cellular and molecular trajectories of pEPs from induction to lineage allocation remains incompletely defined, including how conserved versus species‐specific gene regulatory networks (GRNs) direct pEP specification and determination to distinct endocrine fate allocation. Building on insights from mouse endocrinogenesis, multiple protocols differentiate human pluripotent stem cells (hPSCs) into endocrine cells in vitro [8, 9, 10, 11, 12]. However, limited efficiency, particularly at the endocrine induction stage when pEPs form, remains a major barrier to large‐scale production for cell‐replacement diabetes therapy. In addition, human pluripotent stem cell‐derived islets (SC‐islets) frequently contain substantial numbers of enterochromaffin‐like cells absent from adult human islets. Whether these represent transient intermediates of human endocrinogenesis, as suggested by the identification of a serotonin‐producing (5‐HT^+^/INS^+^/PDX1^+^) fetal β‐cell population that declines postnatally [13], or largely reflect an in vitro‐skewed outcome remains unresolved [14, 15, 16]. These gaps underscore the need for comprehensive, cross‐species comparisons of pEPs across mouse and human, in vivo and in vitro.

Compared to the pancreas, where endocrinogenesis is confined to embryonic and fetal development, the intestine, a larger endocrine organ, generates short‐lived enteroendocrine cells (EECs) continuously throughout adult life. This process begins when intestinal stem cells (ISCs) differentiate into iEPs, which like pEPs, are marked by NGN3 expressions. iEPs then diversify into multiple EEC subtypes, including L, K, I, S, and enterochromaffin cells [17, 18, 19]. Although both organs rely on EPs for endocrinogenesis, comparative profiling of this population across pancreas and intestine remains lacking. Given the potential of intestinal epithelial cells to generate insulin‐producing cells [20, 21, 22], identifying shared and distinct molecular signatures of pEPs and iEPs could inform iEPs reprogramming for β‐cells regeneration or expanding GLP‐1‐producing L‐cells for obesity treatment [5]. This knowledge may also help clarify differences in the kinetics and life span of pancreatic versus intestinal endocrine cell formation [23, 24].

Despite extensive efforts to dissect pancreatic endocrine lineage formation using single‐cell omics [25, 26, 27, 28, 29, 30, 31, 32, 33, 34, 35], pEPs profiling remains incomplete due to their scarcity and lack of efficient isolation tools. We addressed this using our recently reported paired single‐nucleus (sn) RNA‐seq and ATAC‐seq dataset [36] from an Ngn3‐Venus reporter mouse line enabling pEP enrichment [26] combined with human in vivo [37] and in vitro [38] multiomic data and proteomics profiling of pEP‐enriched epithelial cells, to comprehensively characterize human and mouse pEPs. We further extended this paired approach to Ngn3^+^‐enriched mouse intestinal cells, comparing pEPs and iEPs at the transcriptomic and epigenomic levels. Together, our work provides a cross‐species, cross‐organ resource to understand cellular and molecular trajectories of endocrinogenesis.

## 2 Results

### 2.1 SnRNA‐seq/snATAC‐seq Analysis Enables Cross‐Species Profiling of Pancreatic EPs

**FIGURE 1:**
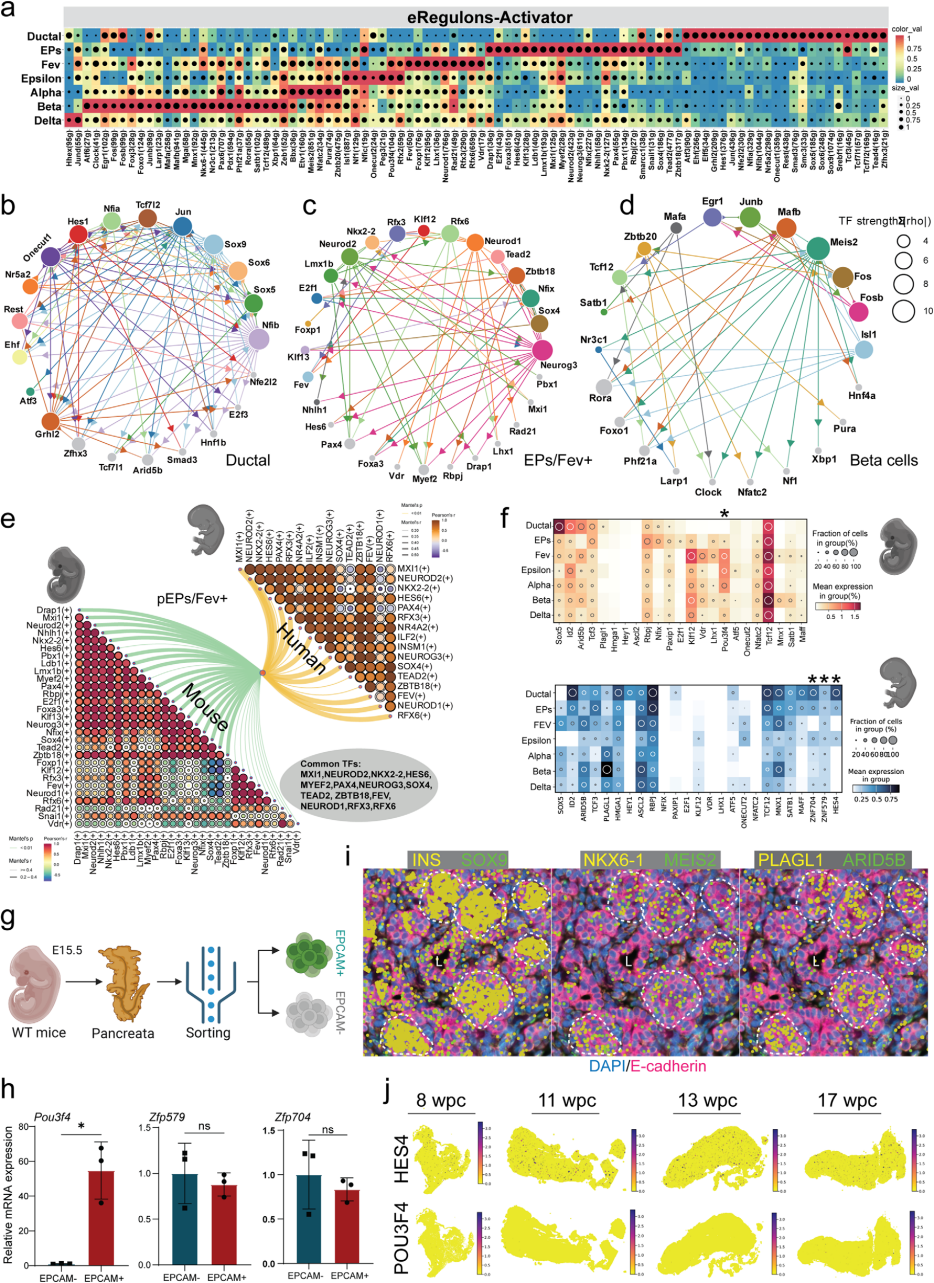
Cross‐species GRNs of the pancreatic endocrine lineage during development. (a) Heatmap of TFs identified as activating in mouse embryonic pancreatic lineages, with colors indicating expression levels and dark circles marking level of activity. (b–d) Circular TF correlation networks of TF‐TF gene regulatory interactions in ductal, EP, Fev^+^, and endocrine cell lineages, with arrowheads indicating downstream targets. Each node represents a TF, node size corresponds to its overall regulatory strength (Σ|ρ|), and node color denotes TF identity (unique colors for activators and grey for target‐only factors). Arrows indicate positive regulatory correlations between TFs, drawn as curved lines placed 8 mm before the target node to minimize overlap. (e) Triangular heatmaps comparing pEP/Fev^+^‐specific TFs between TF‐gene regulatory networks within mouse and human datasets. The heatmap shows Pearson correlations among regulon activities, curved links connect orthologous regulon blocks between mouse and human EPs/Fev^+^ cells, with edge width scaled to Mantel's effect size and color to Mantel's *p* value (*p* < 0.05). (f) Dot plots illustrating distinct expression patterns of TFs not identified as common in SCENIC^+^ analysis between mouse and human. Asterisks indicate genes that were not detected in the other species. (g) Scheme of the FACS‐sorting strategy used to isolate epithelial and non‐epithelial fractions from mouse pancreata. (h) qPCR of sorted mouse embryonic EPCAM− and EPCAM+ fractions. *n* = 3; Data are mean ± SD; two‐tailed t‐test; *p < 0.05; ns, not significant. (i) Representative Xenium spatial transcriptomics images of the developing human pancreas. Endocrine clusters are shown by dashed outlines and SOX9 marks ductal epithelium. L, lumen. (j) Spatial expression of *HES4* and *POU3F4* across developing human pancreas. *HES4* is detected across all stages, whereas *POU3F4* is essentially undetectable throughout.

To elucidate the molecular mechanisms underlying pancreatic endocrinogenesis, we deeply profiled our recently acquired paired single‐nucleus RNA sequencing (snRNA‐seq) and single‐nucleus assay for transposase‐accessible chromatin sequencing (snATAC‐seq) [36]. This mouse pancreatic epithelium dataset, enriched for pEPs, includes samples collected at sequential developmental stages (E14.5, E15.5, E16.5) from the Ngn3‐Venus fusion (NVF) reporter mouse line, which selectively labels Ngn3^+^ cells and enables prospective pEP enrichment [26] (Figure S1a). Because endocrine differentiation arises within the pancreatic epithelium, the dataset included both endocrine and ductal populations (15186 total cells, including 5479 EPs) yielding seven clusters: ductal, EPs (Ngn3^low^, Ngn3^high^, ε progenitors), Fev^+^, α‐lineage (Fev^+^ pre‐α and α‐cells), β‐lineage (Fev^+^ pre‐β and β‐cells), δ‐lineage (Fev^+^ pre‐δ and δ‐cells), and ε‐cells (Figure S1b‐d). To define enhancer‐driven GRNs during endocrinogenesis in vivo, we applied SCENIC^+^, which predicts enhancers, their upstream TFs, and target genes [39] (Figure S1e). This analysis identified 91 putative activating TFs (≥15 target genes) across all lineages (Figure 1a; Table S1).

Using TF expression and regulon (TF‐region‐gene link) activity (Figure S1f) we identified key TFs within each cluster (Table S2a) and mapped their interactions. Ductal GRN included canonical TFs (e.g., *Sox9*, *Hes1*, *Rest*), alongside less‐ characterized regulators (e.g., *Grhl2*, *Arid5a*, *Nr5a2*) (Figure 1b). EP GRNs featured known genes (e.g., *Ngn3*, *Nkx2‐2*, *Pax4*) alongside underexplored TFs (e.g., *Zbtb18*, *Nfix*, *Mxi1*) (Figure 1c), and the Fev^+^ GRN combined established (e.g., *Fev*, *Neurod1*, *Rfx6*) and novel (e.g., *Klf12*, *Lcorl*, *Prdm16*) TFs (Figure 1c). β‐cell GRNs exhibited a rich set of TFs, combining well‐studied (e.g., *Pdx1*, *Nkx6‐1*, *Mafa*) with less‐characterized factors (e.g., *Meis2*, *Zbtb20*, *Nr3c1*) (Figure 1d). Other endocrine lineages contained far fewer TFs by comparison (Table S2a). Combining the target genes of all cluster‐specific TFs (Figure S2a), we performed gene ontology analysis and canonical signaling pathway analysis to resolve cluster‐specific programs and their inferred activity (Table S2b‐f). Ductal target genes drove tube morphogenesis, cell junction and cell projection organization, mainly regulated by Sox9 and Nfib, while EP genes converged on translation, axon guidance and planar cell polarity, predominantly under Ngn3, Tead2 and Sox4 regulation. Fev^+^ genes were enriched for vesicle‐mediated transport and regulation of secretion, and β‐cell genes for regulation of insulin secretion, ER stress response and exocytosis, mainly regulated by Pdx1, Mafb and Meis2 (Figure S2b). Signaling pathway enrichment across the four states mirrored their developmental positions. Ductal cells showed the broadest enrichment, dominated by PI3K‐Akt, focal adhesion and morphogenetic signaling including Wnt, Hippo and BMP, whereas endocrine progenitors displayed comparatively sparse pathway activity. Fev^+^ cells were enriched for GPCR, cAMP and hormone‐mediated signaling, and β‐cells for insulin, metabolic and broader hormonal pathways. As these terms derive from curated pathway gene sets that may include broadly expressed genes, they reflect enrichment associations rather than activated signaling and are consistent with a progressive shift in signaling context along the endocrine trajectory (Figure S2c; Table S2g).

To compare TFs across species, we analyzed unpaired scRNA‐seq (week 8–12) and sn‐ATAC‐seq (week 12) datasets from human embryonic/fetal pancreatic epithelium (682 pEPs in scRNA‐seq and 198 in sn‐ATAC‐seq) [37]. SCENIC^+^ identified 84 active human TFs (Figure S3a; Table S3), revealing shared and species‐specific factors: 11 TFs (e.g., *SOX9*, *REST*, *HES1*) were conserved in the ductal cluster (Figure S3b), 14 in EP/FEV^+^ clusters (e.g., *NGN3*, *TEAD2*, *NKX2‐2*) (Figure 1e), and 11 in endocrine cells (e.g., *PDX1*, *NKX6‐1*, *MEIS2*) (Figure S3c). Notably, TFs not classified as shared often showed similar expression patterns across species (Figure S3d), yet clear differences also emerged. For instance, *ZNF704* and *HES4* were detected only in human, whereas *Pou3f4* was detected only in mouse. *HEY1* and *ASCL2* were high in human but weak in mouse, while *NFIX* and *LHX1* showed a reverse pattern. Some TFs also shifted cell‐type identity between species. For instance, *MAFF* and *SATB1* were active in human ductal cells but mouse endocrine cells, whereas *SOX5* and *PLAGL1*, duct‐specific in mice, were enriched in human endocrine lineages (Figure 1f).

To validate these species‐specific patterns, we FACS‐sorted epithelial (EPCAM^+^) and non‐epithelial (EPCAM^−^) fractions from E15.5 mouse pancreata [26] (Figure 1g), confirming separation by qPCR (Figure S4a). *Pou3f4* mRNA was epithelium‐enriched, whereas *Zfp579* and *Zfp704* (mouse orthologs of *ZNF579/ZNF704*) showed no such enrichment (Figure 1h), supporting species‐specific divergence. To further validate these patterns in an independent human dataset, we leveraged a previously generated single‐cell atlas of the developing human pancreas [40]. Clustering resolved ductal, progenitor, and endocrine populations, in which our species‐specific and divergent TFs, as well as the canonical EP‐specific TFs, showed the expected cluster‐restricted expression, consistent with our human analysis (Figure S4b‐d). We next examined the spatial context of these TFs using a spatial transcriptomics dataset of developing human pancreas spanning 8, 10–14, and 17 post‐conceptional week (PCW) [41] (Figure S5a,b), which recapitulated most TF expression patterns from our human analysis (Figure S5c), with canonical EP‐specific TFs enriched in the EP cluster (Figure S5d). Xenium imaging confirmed correct cluster identification, with *INS*, *NKX6‐1*, and *MEIS2* enriched in endocrine clusters and *SOX9* marking ductal epithelium (Figure 1i). *PLAGL1* was clearly enriched in endocrine cells, corroborating its human‐specific endocrine expression despite being duct‐specific in mouse (Figure 1i). Finally, *HES4* (which lacks a mouse ortholog) [42] and *HEY1* were detected across all stages, while *POU3F4* was essentially undetectable at every stage (Figure 1j; Figure S5e). Together, these orthogonal mouse epithelial‐sorting and independent human single‐cell and spatial datasets confirm that species‐specific TF assignments reflect genuine biological divergence rather than technical or sampling artifacts.

### 2.2 PEPs Express a Set of Unique Highly Enriched Genes (HEGs) Associated With Glycemic Traits

**FIGURE 2:**
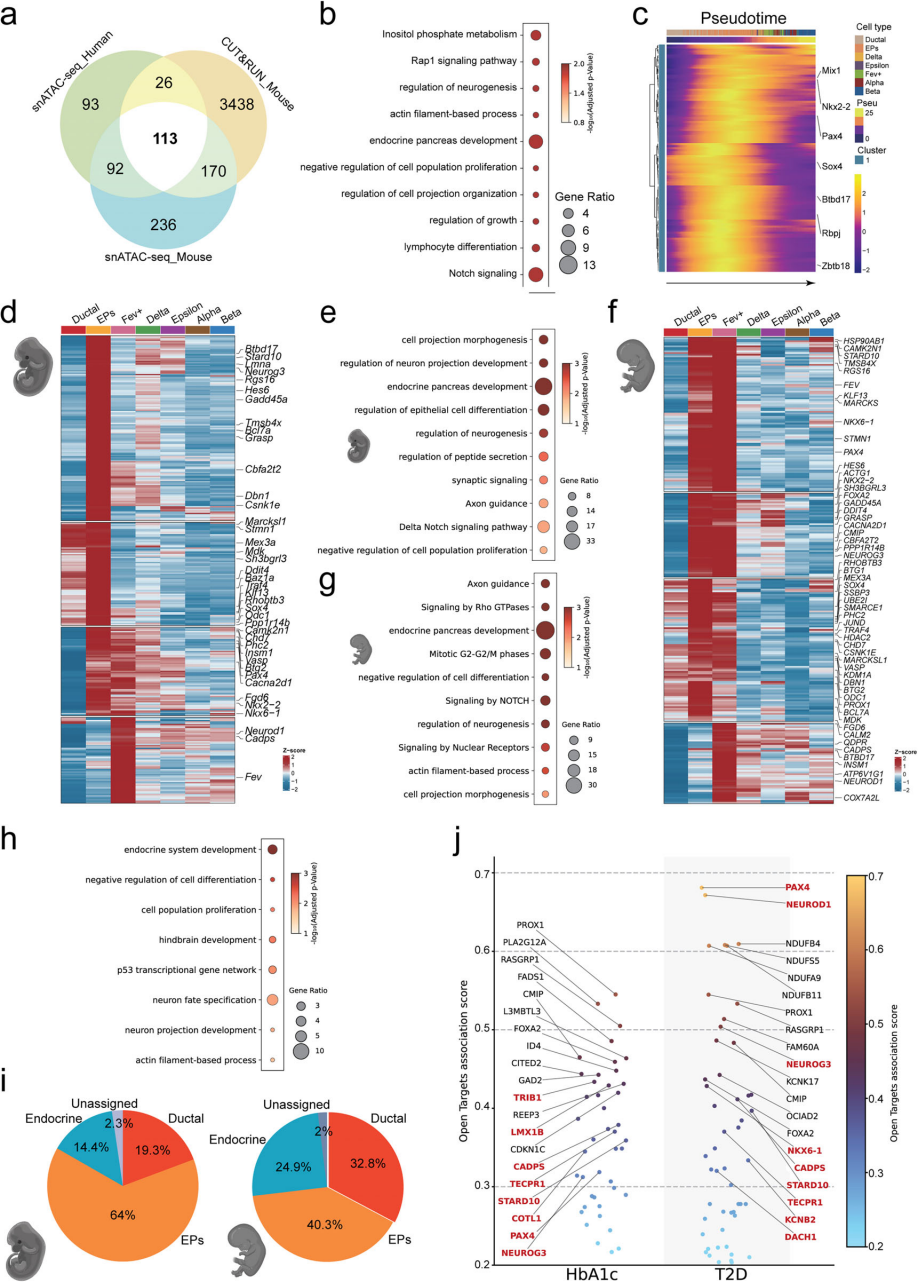
Cross‐species profiling of pEPs. (a) Venn diagram of NGN3 targets in pancreas, identified from mouse and human snATAC‐seq data and from in vivo mouse pancreas CUT&RUN analysis. The list of genes is provided in Table S4b. (b) Pathway enrichment analysis of the 113 common NGN3 targets identified in (a). Selected terms are shown. The list of all terms is provided in Table S4c. (c) Pseudotime plot illustrating peak expression of the 113 common targets in EPs across mouse endocrinogenesis. Arrow shows directionality. (d) Heatmap of 350 HEGs in mouse pEP/Fev^+^ clusters. (e) Pathway enrichment analysis of genes identified in (d). Selected terms are shown. The list of all terms is provided in Table S5b. (f) Heatmap of 275 HEGs in human pEP/FEV^+^ clusters. (g) Pathway enrichment analysis of genes identified in (f). Selected terms are shown. The list of all terms is provided in Table S5d. (h) Pathway enrichment analysis of common HEGs genes identified in mouse and human. Selected terms are shown. The list of all terms is provided in Table S5e. (i) Pie charts showing the distribution of TFs by cluster that predominantly regulate HEG expression. (j) Association of human pEP HEGs with glycemic traits. Common detected HEGs between mouse and human are labeled in red.

We next compared TF target genes across species. While a few TFs shared substantial numbers of targets, most showed limited overlap, indicating species‐specific regulation (Table S4a). Given its master regulatory role in endocrine cell induction, NGN3 was analyzed in greater depth. Cross‐species comparison of NGN3 targets identified 205 shared genes (Figure 2a; Table S4b). Integration with Ngn3 CUT&RUN data from embryonic mouse Ngn3^+^ cells [43] confirmed direct binding at 113 of these targets (Figure 2a). These conserved targets are enriched in pathways related to Notch signaling, proliferation, differentiation, and actin cytoskeleton organization (Figure 2b; Table S4c). They include transcriptional activators (e.g., *NKX2*‐2, *PAX4*) and repressors (e.g., *CBFA2T2*, *RCOR2*), cytoskeletal regulators (e.g., *ACTN3*, *DBN1*), cell cycle mediators (e.g., *CDK2AP1*, *GADD45A*), and signaling components (e.g., *INPPL1*, *SEMA3G*). In mouse, many of these NGN3 targets peak in expression in pEPs, suggesting temporally specific roles in endocrine lineage specification (Figure 2c).

The transient nature of pEPs and FEV^+^ cells likely reflects temporary activation of molecular programs important for their development. We conducted differential gene expression analysis and identified 350 highly enriched genes (HEGs) (logFC ≥1.5) in mouse pEPs/Fev^+^ cells, with very low or negligible expression in other pancreatic lineages (Figure 2d; Table S5a). Pathway enrichment indicated association of these genes with neural differentiation, tissue morphogenesis, Notch signaling, and cell division (Figure 2e; Table S5b). The same analysis in human data revealed 275 HEGs (Figure 2f; Table S5c), enriched for cell cycle, endocrine development, morphogenesis, and cytoskeleton (Figure 2g; Table S5d). Cross‐species comparison identified 55 shared HEGs between mouse and human, including transcriptional regulators of neuroendocrine differentiation (e.g., *NGN3*, *NEUROD1*, *PAX4*), morphogenetic genes (e.g., *TRAF4*, *MDK*, *DACH1*), and actin cytoskeletal regulators (e.g., *DBN1*, *TMSB4X*, *VASP*) (Figure 2h; Table S5e). These conserved genes are predicted to be predominantly regulated by pEP‐specific TFs in both species, underscoring their potential roles in pEP induction and endocrine lineage specification (Figure 2i).

We next examined human HEG association with the glycemic traits HbA1c and T2D using the Open Targets Platform (https://platform.opentargets.org) [44], focusing on these traits as the phenotypes most directly attributable to β‐cell function and endocrine hormone regulation. In total, 43 genes were significantly associated with at least one trait (score ≥0.3), and 12 shared between human and mouse (Figure 2j; Table S5f) including *NGN3* and *PAX4*, previously implicated in delayed‐onset and transient neonatal diabetes, respectively [45, 46, 47, 48]. Several genes, such as the homeobox TF *PROX1*, the lipid/phosphoinositide transfer protein *STARD10*, the signaling adaptor *CMIP*, and the Ca^2^^+^‐regulated exocytosis factor *CADPS*, showed associations with both traits (Figure 2j). To determine whether these associations reflect adult β‐cell function or endocrine development, we examined the expression of the 43 genes in adult human islets (accession number: GSE198623) [49]. While most were expressed at varying levels, 8 were not detected in the adult islet scRNA‐seq (*ARF5*, *COTL1*, *DACH1*, *FAM60A*, *MARCKS*, *NUDT4*, *SRP9*, *ZNRF1*) and a further 6 showed only negligible expression (Figure S5f), suggesting their trait association likely stems from roles in endocrine cell formation rather than adult islet function.

### 2.3 Human in Vivo Pancreatic EPs Show a Distinct Molecular Profile Compared to In Vitro SC‐EPs

**FIGURE 3:**
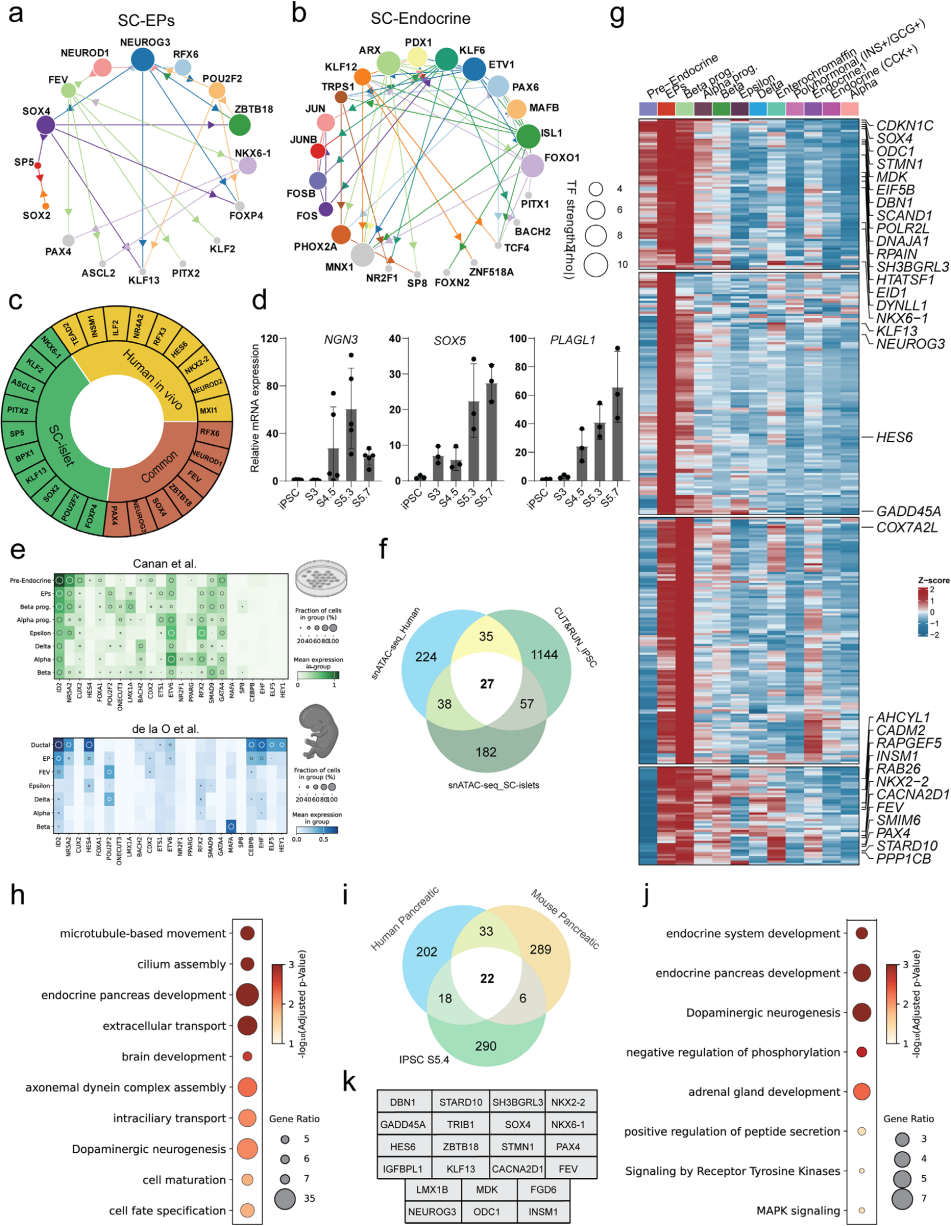
Cross‐system mapping of GRNs in the pancreatic endocrine lineage and pEP profiling. (a,b) Circular TF correlation networks of GRNs in SC‐EPs and SC‐endocrine cells during human in vitro differentiation; arrows indicate downstream targets. (c) Common and differentially detected TFs active in pEP/FEV^+^ cells in human in vitro and in vivo systems. (d) qPCR analysis of *SOX5* and *PLAGL1* mRNA expression during SC‐islet differentiation. (e) Dot plots illustrating distinct expression patterns of TFs not identified as common in SCENIC^+^ analysis between human in vivo and in vitro systems. (f) Venn diagram of NGN3 targets identified from human in vivo and in vitro multiomics data and from in vitro SC‐islet CUT&RUN analysis. (g) Heatmap of 336 HEGs in SC‐EP/FEV^+^ clusters in vitro. (h) Pathway enrichment analysis of genes identified in (g). Selected terms are shown. The list of all terms is provided in Table S7d. (i) Venn diagram of common HEGs between mouse and human in vivo samples and SC‐islets. 22 common genes between the three datasets are shown. (j) Pathway enrichment analysis of common HEGs between human in vivo and in vitro. Selected terms are shown. The list of all terms is provided in Table S7e. (k) List of 22 HEGs genes detected as common between mouse and human in vivo and SC‐islets.

SC‐islets do not fully recapitulate adult human islets in maturity and functionality, owing to differences between in vivo development and in vitro differentiation. To assess how closely stem cell‐derived EPs (SC‐EPs) align with in vivo human pEPs, we applied SCENIC^+^ to a previously generated EP‐enriched population sorted at stage 5 of differentiation (S5), the peak of SC‐EP formation, from the ARX^CFP/CFP^/PAX4^mCherry/mCherry^ reporter cell line (including 1524 EPs) (Figure S6a‐c) [38]. This identified 93 TFs (Figure S6d), including 16 in SC‐EPs and 23 in SC‐endocrine cells (Figure 3a,b; Table S6). Cross‐referencing with in vivo data revealed only 7 common TFs in EPs (e.g. *NGN3*, *FEV*, *PAX4*, *ZBTB18*) (Figure 3c). However, despite differential detection in the SCENIC^+^ analysis, many TFs showed comparable expression patterns across lineages in both systems (Figure S6e). Consistent with both human in vivo profiles, *SOX5* and *PLAGL1 (ZAC1)*, an imprinted transient neonatal diabetes candidate [50], were upregulated in endocrine cells in the in vitro dataset (Figure S6e). qPCR in SC‐islets further validated induction of both genes at endocrine‐lineage stages, highlighting conserved expression patterns in human systems but divergent profiles in mouse (Figure 3d). Conversely, TFs including *EHF*, *GATA4*, *MAFA*, *MAFF*, *CDX2* and *ETV6* showed distinct lineage‐specific expression between systems (Figure 3e), underscoring differences between in vivo organogenesis and stem cell differentiation.

We then compared target genes of all TFs identified in both systems by SCENIC^+^ to assess shared versus distinct regulation (Table S7a). Focused NGN3 targets analysis identified 65 common genes. Mapping this list onto NGN3 CUT&RUN data from SC–islets in vitro [51] narrowed this set to 27 candidate targets (Figure 3f; Table S7b). Of these, nine genes (*ACTN3*, *CHD7*, *GADD45G*, *KLF13*, *NKX2*‐2, *PAX4*, *SOX4*, *ZBTB18*, *PARD3*) overlapped with mouse in vivo Ngn3 targets (Figure 2a), underlining their conserved role in EP regulation.

To further compare pEPs transcriptional profiles in vivo and in vitro, we identified HEGs in SC‐EPs from transcriptomic data, uncovering 336 genes (Figure 3g; Table S7c) associated with microtubule cytoskeleton, neurogenesis, and protein transport (Figure 3h; Table S7d). Comparison with in vivo human pEP HEGs revealed an overlap of 40 genes, prominently linked to endocrine differentiation, peptide secretion, and cell signaling (Figures 3i,j; Table S7e). Of these, 22 were also shared with mouse pEP HEGs, further suggesting their role in pancreatic endocrinogenesis across systems and species (Figure 3k).

### 2.4 Paired Multi‐Omics Analysis Allows Comparative Profiling of EPs Across Pancreas and Intestine

**FIGURE 4:**
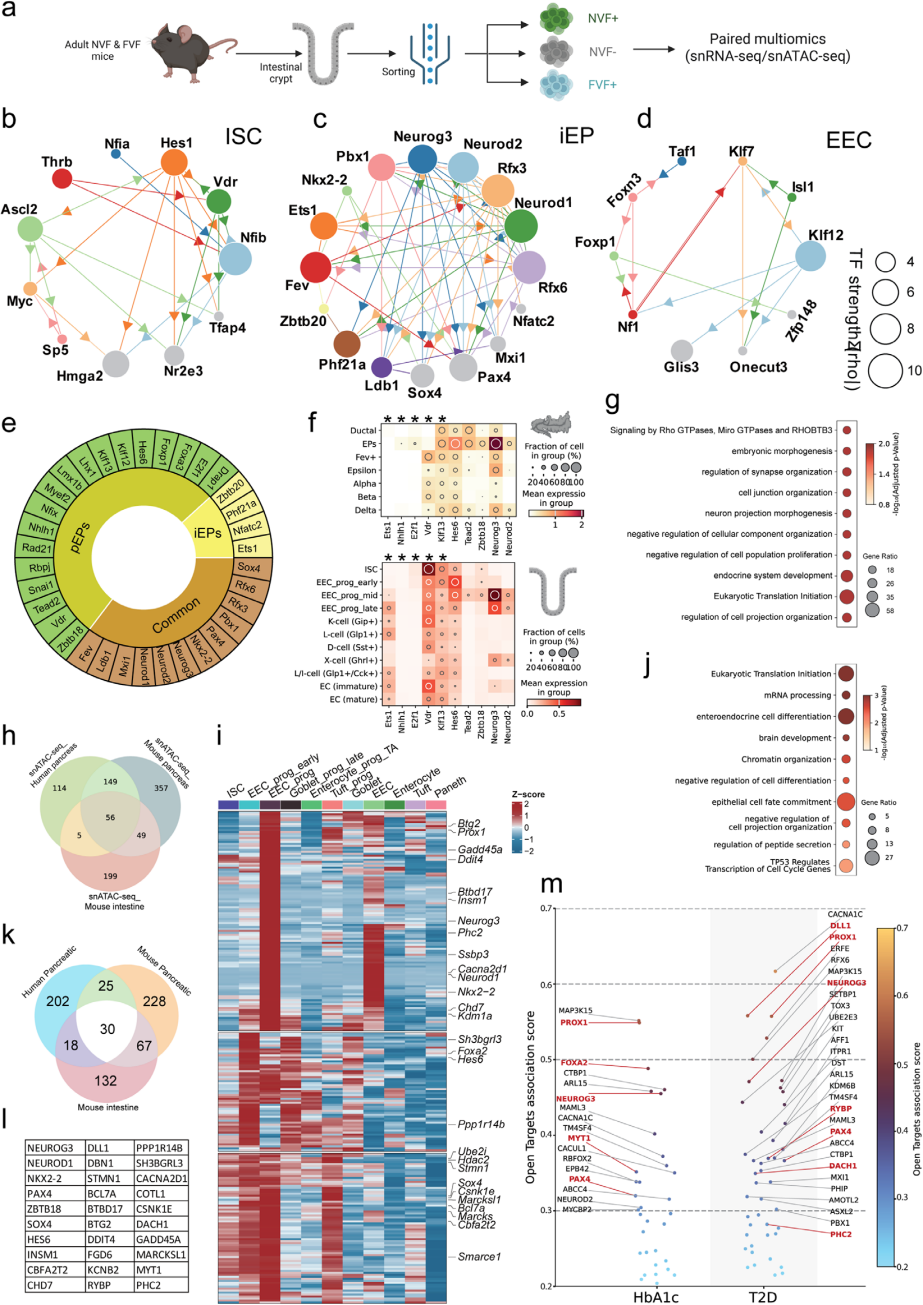
Cross‐organ delineation of GRNs in the endocrine lineage and EP profiling. (a) Schemes show the procedure of performing multiomics of iEP‐enriched intestinal epithelial cells. (b‐d) Circular TF correlation networks of GRNs in intestinal ISCs, EPs, and EECs; arrows indicate downstream targets. (e) Common and differentially detected TFs active in iEPs in mouse pancreas and intestine in vivo. (f) Dot plots illustrating distinct expression patterns of TFs not identified as common in SCENIC^+^ analysis between mouse pancreatic and intestinal endocrinogenesis. Asterisks indicate genes that are expressed differently in the other organ. (g) Pathway enrichment analysis of the 522 commonly expressed targets of progenitor‐specific TFs in mouse pancreas and intestine. Selected terms are shown. The list of all terms is provided in Table S9c. (h) Venn diagram of NGN3 targets identified from mouse and human pancreatic datasets and mouse intestinal multiomics data. (i) Heatmap of 247 HEGs in mouse iEP/Fev^+^ clusters in vivo. (j) Pathway enrichment analysis of the iEP HEGs. Selected terms are shown. The list of all terms is provided in Table S9g. (k) Venn diagram of common HEGs between mouse and human pEPs and mouse iEPs. 30 common genes between the three datasets are shown. (l) List of 30 genes detected as common HEGs between mouse and human pEPs in vivo and mouse intestinal iEPs. (m) Association of mouse iEP HEGs with glycemic risk. Common detected HEGs between human pancreas and mouse intestine are labeled in red.

To compare cross organ endocrinogenesis, we performed paired single‐nucleus multiomics on iEP‐enriched and ISC/secretory‐lineage‐enriched intestinal epithelial cells from adult NVF and Foxa2‐Venus fusion (FVF) mice, respectively (Figure 4a). We profiled 20727 single nuclei (2949 iEPs) forming 11 clusters, including intestinal stem cells (ISCs), enterocyte lineage, tuft cell lineage, goblet and iEP progenitors, goblet cell lineage, Paneth cells, and EEC lineage (Figure S7a,b). Chromatin accessibility at canonical lineage‐marker genes was restricted to the expected clusters, confirming the quality of the snATAC‐seq data (Figure S7c). SCENIC^+^ analysis identified 121 activating TFs across these lineages (Figure S7d; Table S8) and enabled reconstruction of lineage‐specific GRNs (Figure 4b‐d; Figure S7e‐h; Table S9a). Within the EEC lineage, we identified 16 key TFs for iEPs and 10 TFs for EECs (Figure 4c,d). Although fewer TFs were detected in iEPs than mouse pEPs/Fev^+^ cells, most including *Ngn3*, *Fev*, *Nkx2‐2*, *Sox4*, *Neurod2* and *Pax4*, were common to both organs (Figure 4e). We next examined TFs not classified as shared between mouse pancreas and intestine. To resolve their expression in greater detail, we first re‐clustered EECs into individual hormone‐producing cell types (Figure S8a,b). Comparing embryonic pancreatic ductal cells with intestinal ISCs highlighted distinct TF expression (e.g., *Tfap4*, *Ascl2*, *Thrb*) (Figure S8c). Differences were also evident between pEPs/FEV^+^ and iEPs, as *Nhlh1* and *E2f1* were expressed in pEPs and nearly absent in intestinal cells, whereas *Ets1* was expressed in iEPs and scarcely detected in pancreatic lineages. Furthermore, *Vdr* and *Klf13*, which were expressed in pEPs/FEV^+^, showed divergent patterns across intestinal lineages (Figure 4f). Comparing pancreatic endocrine‐specific TFs with intestinal EECs revealed additional contrasts. Consistent with human in vivo and in vitro pancreatic data, *Sox5* was expressed in EECs, while key islet TFs (*Mafa*, *Mafb*, *Meis2*) showed only subtle expression in EECs. Notably, *Pou3f4* was not detected in the intestinal dataset (Figure S8d). Together, these analyses identify multiple lineage‐resolved GRN differences between pancreas and intestinal endocrinogenesis.

We then compared the target genes of all key TFs in iEPs and mouse pEPs/Fev^+^ cells, identifying 522 shared targets (Table S9b), enriched in pathways regulating cell morphogenesis (e.g., *Bmpr1a*, *Plxnb1*), protein synthesis (including several ribosomal protein genes such as Rpl and Rps family members), cytoskeletal dynamics (e.g., *Dbn1*, *Marcks*), and cell cycle inhibition (e.g., *Btg2*, *Cdkn1a*) (Figure 4g; Table S9c). Analysis of shared Ngn3 targets between pEPs and iEPs (Table S9d) identified 105 common genes, 56 of which also overlapped with human in vivo NGN3 targets (Figure 4h). These genes are primarily involved in neuroendocrine development, morphogenesis, actin cytoskeletal organization, and PIP3 signaling (Figure S8e; Table S9e).

To further characterize iEP identity, we used transcriptomic data and identified 247 HEGs (Figure 4i; Table S9f), mainly linked to mRNA processing and translation, enteroendocrine development, epithelial differentiation, and peptide secretion (Figure 4j; Table S9g). Comparison with mouse pEP HEGs revealed 97 shared genes (Table S9h), including regulators of endocrine development (e.g., *Neurod1*, *Rfx6*, *Pax4*), neurogenesis (e.g., *Dll1*, *Kit*, *Ptprs*), and cell projection (e.g., *Cyth2, Dpysl5, Rap1gap2*) (Figure 4k; Figure S8f; Table S9i). Of these, 30 were also common with human in vivo pEP HEGs (Table S9h), including well‐established regulators of endocrine development, such as *NGN3*, *PAX4*, *NEUROD1*, and *NKX2‐2*, among others. We also identified genes with little or no previously reported function in endocrinogenesis, including *ZBTB18*, *BTBD17*, *CSNK1E*, and *SH3BGRL3*, highlighting potentially conserved roles in endocrine formation across species and organs (Figure 4l).

Next, we analyzed associations between iEP HEGs and glycemic traits, identifying 35 genes linked to at least one trait (score ≥0.3) (Figure 4m; Table S9j). From these, 9 were shared between human pEP and intestinal iEP HEGs. These include transcription factors such as *NGN3*, *PAX4*, *PROX1* and *FOXA2*, indicating that variation in these regulators likely affects development of both pancreatic and intestinal endocrine systems, contributing to diabetes risk.

### 2.5 Integrative Analysis Uncovers the Regulatory Programs Driving SC‐EC Formation In Vitro

**FIGURE 5:**
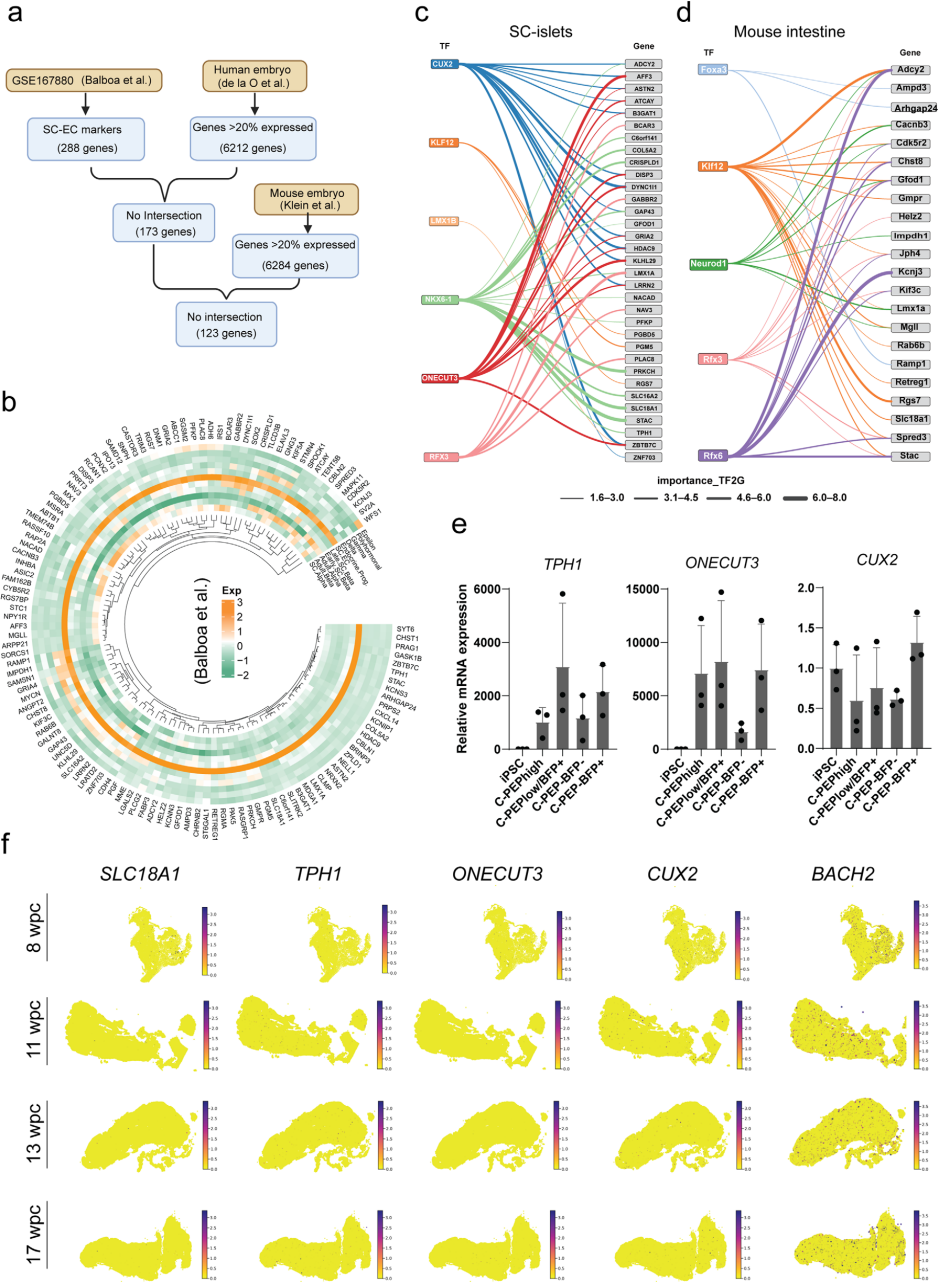
Regulatory drivers of in vitro SC‐EC differentiation. (a) Schematic illustrating the integrative approach used to identify SC‐EC drivers across different datasets. (b) Circular plot displaying the expression of SC‐EC highly expressed genes across different lineages during in vitro SC‐islet differentiation. (c) Sankey plot highlighting the major TFs with the highest number of SC‐EC‐specific target genes during human pancreatic endocrine cell differentiation in vitro. (d) Sankey plot highlighting the major TFs with the highest number of SC‐EC‐specific target genes during mouse intestinal EEC differentiation in vivo. (e) qPCR of *TPH1*, *ONECUT3*, and *CUX2* across sorted populations from the dual‐reporter iPSC line (C‐PEP‐mCherry; SLC18A1‐H2B‐BFP) at S6.14. *n* = 3; Data are mean ± SD. (f) Spatial expressions of *SLC18A1*, *TPH1*, *ONECUT3*, *CUX2*, and *BACH2* in the developing human pancreas.

SC‐derived enterochromaffin cells (SC‐ECs) are enriched during SC‐islet differentiation but largely absent from adult islets. Whether they reflect a genuine developmental state or an in vitro‐skewed program remains debated [13]. To identify potential drivers of SC‐EC differentiation, we first profiled genes highly expressed in SC‐ECs relative to other in vitro cell types, using the scRNA‐seq from a study with sufficiently large SC‐EC population [52] (Table S10a). Cross‐referencing with in vivo mouse and human pancreatic transcriptomic profiles revealed 123 genes specifically enriched in SC‐ECs but absent or minimally expressed in vivo (Figure 5a; Table S10b). As expected, most were strongly or exclusively expressed in SC‐ECs (Figure 5b).

We next examined upstream TFs predicted to regulate SC‐EC‐specific genes, leveraging our recent paired multi‐omics analysis of SC‐islets [38]. Surprisingly, many targets were controlled by TFs traditionally associated with pancreatic endocrinogenesis [37], including NKX6‐1, MNX1, PAX4, ISL1, FEV, and RFX3/6, most also expressed in the gut [17]. In addition, we identified regulators with little or no previously described roles in this context, such as *CUX2*, *CDX1*, *ONECUT1/2/3*, *PHOX2A*, *POU2F2*, and *SP8* (Table S10c). Across both groups, *CUX2*, *NKX6‐1*, *ONECUT3*, *LMX1B*, *RFX3*, and *KLF12* had the most predicted targets within the SC‐EC‐specific gene set (Figure 5c). Reassuringly, many SC‐EC–enriched genes defined from the previously generated dataset [52] were also detectable in our own progenitor‐enriched SC‐islet data [38], indicating the program is reproducible across protocols rather than dataset‐specific. Extending the analysis to in vivo intestinal EEC lineages revealed Klf12, Neurod1, Foxa3, and Rfx3/6 as the TFs with the highest number of targets (Figure 5d). Several TFs, including *ASCL2*, *ATF3*, *FEV*, *FOS*, *ISL1*, *KLF2/5/12*, *NEUROD1*, *NKX2‐2*, and *RFX3/6*, overlapped between in vivo intestinal EECs and SC differentiation (Table S10d). Yet only four TF‐target pairs, including *KLF12‐SPRED3*, *KLF12‐RGS7*, *KLF12‐GFOD1*, and *RFX6‐ADCY2*, were shared between them. Finally, comparing SC‐EC drivers across mouse and human datasets revealed several TFs (e.g., *Klf5*, *Sp5*, *Pou2f2*, *Ascl2* in mouse; *HOXA3*, *TRPS1*, *CUX2*, *SP8* in human) that are absent or very lowly expressed during in vivo pancreatic development (Figure S9a‐c).

To directly test whether these candidate regulators are enriched in cells adopting an EC‐like fate during SC‐islet differentiation, we used a dual‐reporter iPSC line (C‐PEP‐mCherry; SLC18A1‐H2B‐BFP) enabling prospective isolation of SC‐β and SC‐ECs at S6.14 (Figure S10a). Cells were enriched for C‐PEP status: C‐PEP^high^ cells were BFP^−^, whereas C‐PEP^low^ cells were BFP^+^. C‐PEP^−^ cells were further sorted into SLC18A1‐BFP^+^ and SLC18A1‐BFP^−^ populations. By qPCR, *TPH1* and *LMX1B* expression closely tracked SLC18A1‐BFP status across all populations, while *ONECUT3* and *CUX2* followed a less clear pattern. Both genes were expressed across C‐PEP^+^ populations but slightly higher in the C‐PEP^low^ BFP^+^ than C‐PEP^high^ BFP^−^ fraction, and higher in the SLC18A1‐BFP^+^ fraction within C‐PEP^−^ cells. In contrast, *HDAC9*, *TRPS1*, and *BACH2* showed a less consistent enrichment across the sorted populations (Figure 5e; Figure S10b).

We next asked whether candidate SC‐EC drivers are also expressed during in vivo human pancreas development, using the spatial (Xenium) dataset spanning 8–17 wpc, which covers the stages EP and immature islet cell formation. *SLC18A1*, *TPH1*, *ONECUT3*, *CUX2*, *POU2F2*, and *LMX1B* were not detected at any stage (Figure 5f; Figure S10c), contrasting with their robust co‐expression in SC‐ECs in vitro. By contrast, *TRPS1*, *BACH2* and *HDAC9* showed only low, sparse signal across all stages examined (Figure 5f; Figure S10c). Thus, the large majority of the SC‐EC‐associated regulatory program is not recapitulated in vivo, with only a small subset of markers genes overlapping.

To test whether CUX2 and ONECUT3 functionally direct EC differentiation, we performed in silico perturbation analysis on the SC‐islet dataset. Neighborhood composition and marker expression defined Pre‐Endocrine, EPs, β‐progenitor, and SC‐ECs as a coherent lineage culminating in the EC fate, within which Palantir pseudotime identified SC‐ECs as the sole terminal state (Figure S11a,b). CellOracle virtual knockout of either factor reduced directional differentiation toward the EC fate, with negative perturbation scores concentrated at Pre‐Endocrine and EP grid points (Bonferroni‐corrected *p* < 0.005) and absent in randomized GRN controls (Figure S11c‐e). Markov simulations further showed that loss of either factor redistributed predicted fates toward the α‐lineage at the expense of the EC fate (Figure S11f,g). These results predict that CUX2 and ONECUT3 act at the endocrine progenitor stage to sustain differentiation toward the EC fate.

### 2.6 Cross‐Species and Cross‐Organ Analyses Reveal Shared and Distinct EP Communication Patterns

To explore direct and indirect cell–cell interactions, we inferred ligand–receptor (L–R) pairs using CellChat [53], identifying ligands and receptors expressed by progenitor populations (pEP/Fev^+^ cells and iEPs) and their neighboring cell types. This yielded 139 L–R pairs in mouse pancreas, 436 in human pancreas, 593 in human in vitro differentiation, and 58 in mouse intestinal cells (Table S11a‐d). Mouse and human pancreatic progenitors shared several L–R interactions involving key developmental pathways, with an even broader overlap between in vitro and in vivo human datasets. Several pairs were conserved across all three pancreatic datasets, including *AGRN–DAG1*, *FGF9–FGFR3/4*, *GRN–SORT1*, and *SEMA6A–PLXNA2* (Figure S12a). In contrast, cross‐organ comparison between mouse pancreatic and intestinal progenitors revealed only a few shared interactions, including homophilic *MPZL1–MPZL1* and *F11R–F11R*, and heterophilic *KIT–KITL*, *LAMB1–ITGB1*, and *LAMA5–ITGB1* (Figure S12a). Notably, *MPZL1–MPZL1* and *LAMB1–ITGB1* were also observed in both human in vivo and in vitro pancreatic datasets, suggesting a conserved role in progenitor communication across organs and species. We then analyzed L–R interactions involving pEPs in mouse and human in vivo and SC‐islets. Among pEP‐expressed ligands were several Notch ligands, including *DLL1* and *DLK1*(Figure S12b), consistent with their role in lateral inhibition that suppresses endocrine differentiation in neighboring bipotent progenitors [54, 55]. Multiple homophilic interactions among pEPs suggested mechanisms for progenitor–progenitor communication (Figure S12b). Notably, pEPs expressed a wider range of receptors than ligands, including Wnt signaling components, FGFRs, integrins, and morphogenetic receptors such as plexins and Kit (Figure S12c).

### 2.7 Proteomic and Transcriptomic Analyses Reveal Shifts in Multiple Cellular Programs During Endocrinogenesis

**FIGURE 6:**
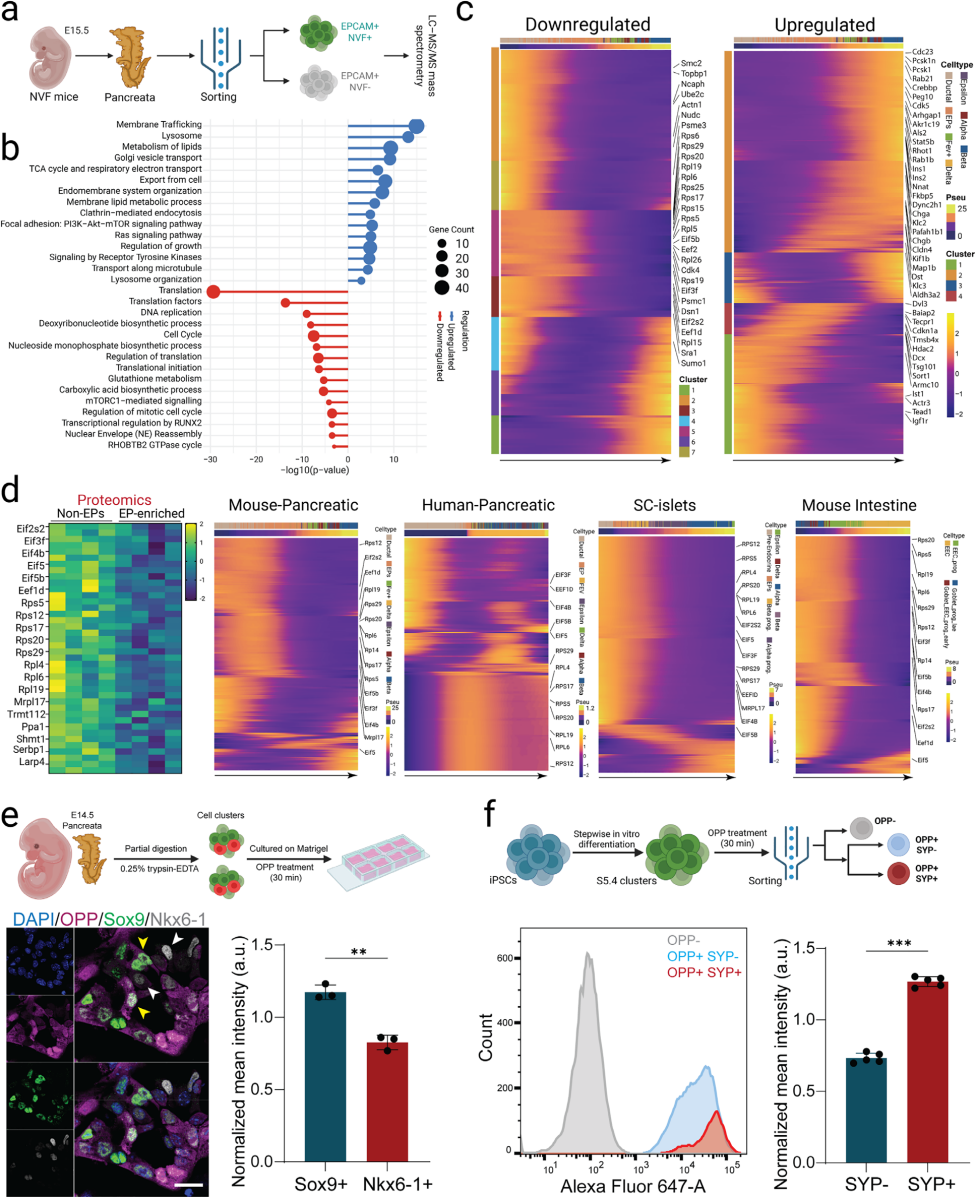
Endocrinogenesis is accompanied by changes in multiple cellular programs. (a) Schematic outlining the sample preparation and proteomics workflow for mouse EP‐enriched pancreas samples. (b) Pathway enrichment analysis of proteins upregulated and downregulated in pEP‐enriched versus non‐EP fractions. 30 selected terms are shown. The list of all terms is provided in Table S12d,e. (c) Pseudotime plot depicting mRNA expression of proteins identified in the proteomics analysis during mouse endocrinogenesis. The x‐axis represents pseudotime units (Peu). Arrows show directionality. (d) Heatmaps and pseudotime plots profiling protein and mRNA expression of translation‐associated genes across datasets. (e) OPP labelling of cultured E14.5 mouse pancreatic clusters. Scheme of the assay is shown on top. Representative confocal images showing OPP staining in Nkx6‐1^+^ (white arrowheads) and Sox9^+^ (yellow arrowheads) cells. Quantification shows significantly higher normalized OPP mean intensity in Sox9^+^ progenitor/ductal cells than in Nkx6‐1^+^ endocrine cells. Scale bar, 20 µm. (f) OPP labelling during in vitro SC‐islet differentiation. Scheme of the assay is shown on top. Representative FACS plot of OPP levels in different cell populations. Quantification shows higher normalized OPP mean intensity in SYP^+^ than SYP^−^ cells. *n* = 3 (e) and 5 (f); Data are mean ± SD; two‐tailed t‐test; **p < 0.01,***p < 0.001; ns, not significant.

To compare the multiome analysis at the protein level, we conducted quantitative proteomics on mouse embryonic pancreatic epithelial cells (Figure 6a). Mass spectrometry quantified over 7,000 proteins from pEP‐enriched (pEPe) versus non‐pEP cells isolated from the NVF reporter mouse line at E15.5. We identified 701 differentially expressed proteins, comprising 198 downregulated and 503 upregulated proteins in pEP versus non‐pEP cells (Table S12a‐c). Pathway analysis linked downregulated proteins primarily to translation, DNA replication, and cell division, whereas upregulated proteins were enriched for cell growth, cytoskeletal dynamics, endomembrane organization, vesicle trafficking, lysosomal activity, lipid metabolism, and diverse signaling cascades (Figure 6b; Figure S13a; Table S12d,e). Together, this indicates a shift toward reduced cell division, increased membrane trafficking and turnover, and cytoskeletal remodeling during endocrine lineage formation.

To align proteomics and multiomics data, we analyzed differentially regulated proteins across scRNA‐seq pseudotime. Downregulated proteins were predominantly expressed in mouse ductal cells, whereas upregulated proteins localized to pEPs, Fev^+^ cells, and endocrine cells. This validated the proteomic data and allowed mapping protein expression to specific cell types at the mRNA level (Figure 6c). We then analyzed mRNA expression of proteins linked to key cellular processes highlighted by proteomics including cell cycle and growth, protein synthesis, and the microtubule (MT) cytoskeleton. Consistent with the proteomics findings, genes linked to cell cycle arrest and growth were upregulated, while mitotic drivers were downregulated during endocrinogenesis across all datasets (Figure S13b,c). Furthermore, genes encoding ribosomal proteins and translation regulators showed either sustained expression in early progenitors or a transient peak in EPs, followed by sharp downregulation in hormone‐expressing cells (Figure 6d). To directly test whether these changes translate into altered protein synthesis activity, we performed O‐propargyl‐puromycin (OPP) labelling, which quantifies nascent translation in individual cells. In cultured E14.5 mouse pancreatic clusters pulsed with OPP for 30 min, Sox9^+^ progenitor/ductal cells showed significantly higher OPP incorporation than Nkx6‐1^+^ endocrine cells (Figure 6e), consistent with the pseudotime downregulation of ribosomal and translation‐regulator genes upon endocrine commitment. The same assay was applied to SC‐islets at 5.4 clusters, pulsing with OPP for 30 min and sorting cells by OPP incorporation and the endocrine marker synaptophysin (SYP). Surprisingly, and in contrast to the in vivo pattern, OPP^+^ SYP^+^ endocrine cells showed higher nascent translation than OPP^+^ SYP^−^ cells (Figure 6f), likely a system‐ or species‐specific difference. Together, these assays functionally confirm that translational activity is dynamically remodelled during endocrinogenesis.

Beyond translational control, endocrinogenesis also accompanied by remodelling of the microtubule (MT) cytoskeleton. Many MT‐related genes increased during endocrinogenesis (Figure S14a), consistent with the heightened secretory demand of endocrine cells and their need for a more elaborate, polarized MT network. Among these, we examined two MT‐associated proteins not been previously reported in pancreatic or intestinal endocrine cells: the MT‐stabilizing adaptor microtubule‐associated protein 1B (MAP1B) and the MT–actin–membrane cytolinker dystonin (DST). Immunostaining confirmed enriched expression of both proteins in endocrine lineages across the mouse embryonic pancreas, adult intestine, and SC‐islets (Figure S14b‐d). Collectively, these findings reveal conserved programs across organs and species, marked by coordinated cell cycle arrest and dynamic remodelling of mRNA translation, alongside upregulation of MT‐associated cytoskeletal components during endocrine lineage formation.

## 3 Discussion

Here, we present a comprehensive multimodal resource that identifies shared and divergent regulatory programs in EPs across species, systems, and organs. By leveraging mouse and iPSC genetic models to enrich this otherwise under‐characterized cell population, our work provides a focused, high‐resolution view of pEP gene‐expression programs and the cellular changes accompanying endocrine lineage acquisition. Unlike prior studies combining scRNA‐seq with bulk or unpaired snATAC‐seq [29, 31], our mouse datasets use paired snRNA‐seq/snATAC‐seq from the same nuclei, directly linking chromatin opening at cis‐regulatory elements to gene expression and enabling more integrated, reliable regulatory predictions [56]. For the human in vivo analysis, paired data were unavailable, so we used scRNA‐seq (pooled across 8–12 pcw) and single‐stage sn‐ATAC‐seq with limited pEP numbers. We, therefore, validated our key species‐specific findings using an independent spatial transcriptomics (Xenium) dataset across multiple developmental stages, though the human predictions should be read with this caveat in mind. A paired multiomic analysis of intestinal epithelial cells enriched for iEPs then enabled direct cross‐organ comparison, deepening our understanding of the shared and distinct mechanisms driving endocrinogenesis in both organs and potentially supporting efforts to reprogram iEPs toward a β‐cell fate [20, 21, 22]. We focused on the adult rather than the embryonic intestine because, unlike the development‐restricted pancreas, the adult gut sustains endocrinogenesis throughout life, the setting relevant to such regenerative strategies.

From these datasets we reconstructed lineage GRNs and recovered most known TFs and their target regulons. Although binding motifs of some TFs were not consistently detected across species or systems, likely reflecting platform limitations, the core TFs defining pEPs and iEPs were identified in every dataset. Importantly, shared expression does not necessarily imply conserved regulatory activity, as a TF may engage different targets, cofactors, or timing in each species. Consistent with this, most shared TFs showed only limited target overlap across species (Table S4a). Similar cross‐species and cross‐organ comparisons, from zebrafish pancreatic and intestinal endocrine cells to pancreatic cell types across distant vertebrates, have likewise found that endocrine identity is preserved through a conserved core of regulators even as their broader networks diverge [57, 58]. Our comparisons should therefore be read as identifying conserved regulatory components rather than fully conserved networks. We also identified several less‐characterized TFs (e.g., *ZBTB18*, *TEAD2*) not previously implicated in endocrinogenesis, a resolution enabled by our NVF reporter line, which substantially increased EP yield and allowed detection of lower‐abundance transcripts. Because the Venus reporter persists beyond active Ngn3 expression, the enriched fraction likely includes some recently Ngn3^+^ cells alongside bona fide EPs. As cell identity here is assigned by transcriptional cluster rather than reporter status, this does not affect our conclusions. Validation of individual species‐specific regulators relied primarily on qPCR and spatial transcriptomics, since several of these understudied factors lack reliable, species‐cross‐reactive antibodies. Therefore, defining the precise roles of these novel regulators through protein‐level analysis and functional loss‐ or gain‐of‐function studies, will be an important next step.

Our enrichment strategy also revealed a set of HEGs whose expression peaks in pEPs/FEV^+^ and iEPs while remaining detectable at lower levels in other lineages. HEGs broaden our earlier EP “signature” set of genes essentially restricted to pEPs, most of which fall within the broader HEG set [26]. Because HEGs were defined by a purely computational threshold, they capture genes enriched in, rather than strictly exclusive to, pEPs/FEV^+^ cells. Some factors with established later roles (e.g., NKX6‐1) retain notable downstream expression yet still pass the cutoff, while genuine EP‐enriched genes just below it are missed. So the HEG set is best read as a sensitivity‐bounded enrichment list, with individual genes interpreted alongside their full profiles in the supplementary tables. Cross‐dataset comparison revealed the highest HEG overlap between mouse pEPs and mouse iEPs, indicating that same‐species in vivo samples share more transcriptional features despite arising from different organs. Human pEPs, by contrast, shared a comparable number of shared HEGs with both mouse pEPs and human SC‐EPs, suggesting that, at least for the differentiation protocol analyzed here, in vivo and in vitro human samples are not necessarily more closely aligned than cross‐species comparisons. Whether this holds across other protocols remains to be determined. Pathway analysis revealed that, beyond differentiation, many in vivo pEPs and iEPs HEGs regulate cellular dynamics and morphogenetic processes. By contrast, HEGs from SC‐EPs showed no such association likely reflecting the requirements for endocrine cell remodeling and morphogenesis [59, 60, 61, 62] that complex in vivo tissue architecture captures more faithfully than simple spheroid cultures.

Many human pEP HEGs were also associated with glycemic traits (HbA1c and T2D). 12 are shared with mouse pEP HEGs, underscoring their evolutionary importance and raising the possibility that their misexpression contributes to glycemic risk. We also found 6 human HEGs that are absent or expressed at very low levels in adult human islets, suggesting that their possible association with glycemic risk may be developmental in origin rather than reflecting adult islet cell function, a hypothesis requiring models with timed genetic alteration. Finally, we identified 9 HEGs shared between the human pancreas and the mouse intestine that are associated with glycemic traits, suggesting impacts on both pancreatic and intestinal endocrine systems. Our analysis may thus help resolve the developmental impact of coding and non‐coding variants, a rapidly expanding area given large‐scale population exome sequencing.

Profiling EPs across datasets allowed us to define genes specific to SC‐ECs and their upstream regulators during stem cell differentiation. Whereas earlier work reported only a few markers of this population in vivo [13], we identified a large number of SC‐EC‐specific genes absent or minimal in both mouse and human in vivo samples. Many of these targets are regulated by TFs well known in pancreatic endocrinogenesis, supporting the view that SC‐ECs and SC‐β‐cells form a continuum of cell states rather than distinct entities at the level of chromatin accessibility [63]. Consistent with this continuum model, although most SC‐EC markers overlap with those of intestinal EECs in vivo, their driver‐target regulatory patterns differ markedly from those governing intestinal EEC development, suggesting SC‐ECs follow a distinct differentiation trajectory. Because this conclusion derives from the specific systems analyzed, it may be influenced by protocol‐ and batch‐related differences and should not be generalized to in vitro islet cells more broadly.

The origin of these cells warrants comment. A recent study reported a transient serotonin‐producing (5‐HT^+^/INS^+^/PDX1^+^) β‐cell population in the fetal and neonatal pancreas that declines after birth [13]. Our data are compatible with limited serotonergic competence in the developing β‐lineage, showing sparse in vivo signal for *TPH1*, *BACH2*, and *HDAC9*. However, the EC‐completion program, including established EC genes such as *SLC18A1* and *LMX1A* and candidate regulators such as *LMX1B*, *ONECUT3*, *CUX2*, and *POU2F2*, is not expressed in vivo at any stage examined. We therefore favor the interpretation that the mature SC‐EC program does not correspond to a cell state present in vivo but is aberrantly activated in vitro, with the transient serotonergic β‐lineage sharing only a small subset of individual markers. This divergence is well illustrated by RFX3, whose loss reduces β‐cells and other islet cells in both species yet diverts them toward different fates. In Rfx3‐deficient mice it expands pancreatic polypeptide^+^ (PP) cells in vivo [64, 65], whereas in human SC‐islets it promotes SC‐EC formation in vitro [66]. Such divergence may reflect less faithful programming of in vitro‐derived cells, genuine species‐specific network differences, or both.

Systematic ligand–receptor (L–R) analysis identified numerous known and several less‐reported interactions across datasets. Notably, pEPs expressed more receptors than ligands, suggesting high responsiveness to microenvironmental signals critical for their induction and lineage specification [67, 68]. Mouse and human pancreatic cells showed relatively high L‐R overlap and the two human datasets overlapped even more, pointing to conserved cell–cell and cell–matrix signaling during endocrine specification in vivo and in vitro. Intestinal cells, by contrast, overlapped less with their pancreatic counterparts, perhaps reflecting differences in cell‐type composition and organ morphology. Identifying pancreas‐specific L–R pairs absent in the intestine may help refine differentiation protocols to steer stem cells toward a pancreatic endocrine fate while limiting off‐target intestinal lineages such as the SC‐ECs described above.

We further present one of the first proteomic datasets of endocrine‐enriched populations during mouse pancreatic development, mapping the mRNA expression of differentially expressed proteins across pancreatic lineages. In both pancreatic and intestinal models, several cellular processes underwent dynamic and opposing changes. A reduction in cell‐cycle inducers alongside increased cell‐growth programs underscore the low proliferative nature of the endocrine lineage in both organs [23, 69]. Additionally, both pancreatic and intestinal endocrinogenesis were marked by downregulation of genes associated with mRNA translation and protein synthesis. Experimentally, we found that the direction of this remodelling differed between two tested systems, with translation declining in mouse endocrine‐committed cells in vivo but increasing in human SC‐islets in vitro, a discrepancy whose basis remains unclear. One likely reason is that the two comparisons are not equivalent. In the mouse, we compare Sox9^+^ ductal cells with endocrine cells, whereas in the SC‐islets we compare endocrine (SYP^+^) cells with SYP^−^ cells, which can be a mixture of different cell types including EPs. This may also reflect that the proliferative trunk epithelium present in vivo might not be present in the stem cell differentiation system. Since modulating protein synthesis enhances β‐cell regeneration in zebrafish [70] and translational control governs stem cell maintenance and fate in other systems such as neural and muscle stem cells [71, 72], its role in EP induction and specification warrants further investigation. Finally, endocrinogenesis was accompanied by increased expression of several microtubule‐related genes and proteins in both organs. This supports our previous findings on increased levels of tubulin isoforms during mouse endocrinogenesis [59], likely reflecting the machinery needed for efficient hormone transport and secretion.

Overall, this EP‐focused resource offers valuable insights into the mechanisms of endocrinogenesis in both the pancreas and intestine. Beyond serving as a key reference for future studies, it provides a detailed comparative analysis of GRNs, HEG genes, and cell–cell communication patterns between progenitor populations in these organs. These findings may also be relevant to other endocrine systems, including the neuroendocrine axis, could ultimately support the effective strategies for generating functional islet cells from stem cells.

## 4 Materials and methods

### 4.1 Mouse Lines and Ethics

This study utilized Ngn3‐Venus fusion (NVF), Foxa2‐Venus fusion (FVF) [73], and CD1 and C57BL/6J wild‐type mice. Animal breeding and sample collection were performed in compliance with relevant legislation and in accordance with the guidelines of the Society for Laboratory Animal Science (GV‐SOLAS) and the Federation of Laboratory Animal Science Associations (FELASA). All experimental procedures were approved by the Helmholtz Munich Animal Welfare Body and the Government of Upper Bavaria (approval numbers ROB‐55.2‐2532.Vet_02‐19‐170 and ROB‐55.2‐2532.Vet_02‐24‐123). Mice were group‐housed (two to four per cage) under controlled environmental conditions (23 ± 1°C; 45–65% humidity) with a 12‐h light/dark cycle and ad libitum access to food and water.

### 4.2 Isolation and Sorting of Single Cells From Murine Small Intestine

To generate ISC‐, iEP‐, and EEC‐enriched datasets, intestinal epithelial cells were processed separately from NVF and FVF reporter mice, enriching for iEPs and for ISCs and secretory lineages including EECs respectively, before combining the resulting datasets for downstream analysis. Small intestines were dissected, cleared of adipose tissue in ice‐cold PBS, opened longitudinally, and stripped of villi and luminal contents using a coverslip. Tissues were cut into ∼2 cm segments, washed in ice‐cold PBS until the supernatant was clear, and incubated in 2 mM EDTA (PBS) for 35 min at 4°C on a benchtop roller. Crypts were released by vigorous shaking in PBS (3–4 rounds), filtered through a 70 µm cell strainer, and pelleted at 300 × g for 5 min at 4°C. For single‐cell dissociation, crypts were incubated in 1–2 mL TrypLE (Life Technologies) for 5 min at 37°C, followed by addition of 5–7 mL complete crypt medium (basal medium + 10% FCS) containing 10 µg/ml DNase I. Cells were incubated for 5–10 min at 37°C with gentle resuspension every ∼2 min, then pelleted (300 × g, 5 min, 4°C) and washed 2–3 times in ice‐cold FACS buffer (1× PBS, 1% FCS, 0.1 mM EDTA). The final suspension was passed through a 40 µm strainer into FACS tubes. Single‐cell suspensions were sorted on a FACS Aria III (BD Biosciences). Cells were gated by FSC‐A and SSC‐A to define the main population, with doublets excluded based on FSC‐W and FSC‐H parameters. Dead cells were removed by SYTOX‐Blue staining (Invitrogen).

### 4.3 Nuclei Isolation and Single‐Cell Multiome Library Preparation

Nuclei were isolated from ∼100k pooled cells using a modified low‐input protocol adapted from 10x Genomics (CG000365). Briefly, sorted cells were washed once with 1 mL PBS containing 1% BSA, counted with Trypan blue staining, and pelleted by centrifugation. The pellet was resuspended in chilled lysis buffer (10 mM Tris‐HCl (pH 7.4), 10 mM NaCl, 3 mM MgCl_2_, 0.01% Tween‐20, 0.01% Nonidet P40 Substitute, 0.005% Digitonin, 1% BSA, 1 mM DTT, and 1 U/µL RNase Inhibitor; 50 µL/sample) and incubated on ice for 4 min. Lysis was quenched by adding wash buffer (500 µL/sample), followed by centrifugation. To transition from wash to diluted nuclei buffer, nuclei were sequentially washed once in a 1:1 mixture of wash buffer and diluted nuclei buffer, then once in pure diluted nuclei buffer. The resulting nuclei pellet was resuspended in 7–10 µL diluted nuclei buffer, and after quality control and counting, immediately processed for single‐cell Multiome library preparation, targeting a recovery of 10,000 nuclei. Libraries were generated using the Chromium Next GEM Single Cell Multiome ATAC + Gene Expression Reagent Bundle (10x Genomics, 1000283) according to the manufacturer's protocol, and sequenced on an Illumina NovaSeq 6000 platform following 10x Genomics recommendations. Raw reads were aligned to the GRCm38 mouse genome (Ensembl release 102) and pre‐processed using Cell Ranger ARC v2.0.2 for downstream analysis.

### 4.4 In Vitro Differentiation of iPSCs (SC‐islets)

Human (HMGUi001) [74] and C‐PEP‐mCherry; SLC18A1‐H2B‐BFP reporter (details will be described in another study) iPSC lines were differentiated into pancreatic endocrine cells in vitro using a previously described protocol [10, 75]. Briefly, iPSCs were used and subjected to a multistep differentiation protocol leading to endocrine and islet cell formation. The stages included definitive endoderm (S1), primitive gut tube (S2), pancreatic progenitor 1 (PP1; S3), pancreatic progenitor 2 (PP2; S4), and endocrine lineage (S5, S6). Cells were routinely tested to confirm the absence of mycoplasma contamination.

### 4.5 RNA Extraction and qPCR Analysis

Total RNA was extracted using the miRNeasy Mini Kit (Qiagen). cDNA synthesis was performed with the SuperScript Vilo cDNA Synthesis Kit (Thermo Fisher Scientific) following the manufacturer's instructions. qPCR was carried out using predesigned TaqMan probes (Life Technologies). Each reaction contained 20 ng cDNA, 4.5 µL nuclease‐free water, 5 µL TaqMan Advanced Master Mix (Life Technologies), and 0.5 µL TaqMan probe (Life Technologies). Reactions were run on a QuantStudio 7 Flex instrument (Thermo Fisher Scientific). GAPDH served as the reference gene for normalization. To preserve data variability and enable statistical analysis under equal variance assumptions, Ct values from iPSC samples were normalized to the average control value. The following primers were used: NEUROG3 (Hs01875204_s1), SOX5 (Hs00374709_m1), PLAGL1 (Hs00414677_m1), and GAPDH (Hs02758991_g1), BACH2 (Hs00935338_m1), TPH1 (Hs00188220_m1), TRPS1 (Hs00936363_m1), Pou3f4 (Mm00447171_s1), Zfp704 (Mm00466953_m1), Zfp579 (Mm02766069_s1), Zbtb18 (Mm02343862_s1), POU2F2 (Hs01552829_m1), ONECUT3 (Hs01369570_m1), CUX2 (Hs00322624_m1), HDAC9 (Hs01081558_m1), LMX1B (Hs00158750_m1), Ins1 (Mm01950294_s1), Nkx6‐1 (Mm00454961_m1), Ngn3 (Mm00437606_s1), Sfrp1 (Mm00489161_m1), Osr1 (Mm00726877_m1), Gli1 (Mm00494654_m1).

### 4.6 Immunostaining and Confocal Microscopy

Embryonic pancreata were fixed in 4% PFA in PBS for 2 hr at 4°C. Fixed tissues were sequentially incubated in 10% and 30% sucrose‐PBS solutions at room temperature (2 h each), followed by equilibration in a 1:1 mixture of 30% sucrose and tissue‐freezing medium (Leica, 14020108926). Samples were then embedded in tissue‐freezing medium to generate cryoblocks, and 10 µm sections were cut using a cryostat. Sections were permeabilized with 0.1% Triton and 0.1 M glycine for 30 min, then blocked for 1 h at room temperature (RT) in blocking buffer (10% FCS, 3% donkey serum, 0.1% BSA, 0.1% Tween‐20 in PBS). Primary antibodies diluted in blocking solution were applied overnight at 4°C. After PBS washes, sections were incubated with secondary antibodies diluted in blocking solution for 3–5 h at RT. Nuclei were counterstained with DAPI for 30 min, followed by PBS washes and mounting in ProLong Gold medium (Life Technologies). Images were captured using a Leica DMI 6000 microscope with LAS AF software or a Zeiss LSM880 inverted confocal microscope. Image analysis was performed with LAS AF and ImageJ software.

The small intestine was isolated from mice, cut into three equal parts, rinsed with PBS, and subsequently fixed as duodenum, jejunum, and ileum (anterior to posterior) in formalin for 3 h at 4°C. After washing with PBS, samples were transferred to sucrose solutions in PBS for 2 h at room temperature at each concentration (7.5%, 15%, and 30%). Tissues were then embedded in OCT (Leica Biosystems, Germany, #14020108926) and stored at −80°C. Sections were cut at 12 µm thickness using a Leica cryostat. Slides were air‐dried for 30 min at room temperature before staining. Staining began with a 1 h incubation in PBS, followed by two rinses with PBST (PBS + 0.1% Tween‐20) and permeabilization for 20 min in 0.1 M glycine, 0.3% Triton X‐100 in H_2_O. After three additional washes with PBST, slides were blocked in DB + 5% BSA for 2 h at room temperature. Primary antibody incubation was carried out overnight at 4°C. Antibodies were diluted in blocking buffer, and 100 µL were applied to each slide. Following incubation, slides were washed twice with PBST. Secondary antibodies were diluted in blocking buffer and incubated on the slides for 2 h at room temperature. Samples were washed twice with PBST, then incubated with Hoechst (1:800 from a 1 mg/ml stock) in PBS for 30 min. After a final wash, 50 µL Elvanol were added, and coverslips were mounted. Imaging was performed using a Zeiss LSM880 confocal microscope in fast AiryScan mode with a 20× dry objective (NA 0.8). Laser lines used were 405 nm, 488 nm, and 546 nm, with a bandpass filter to eliminate channel bleed‐through.

For SC‐islet immunostaining, cryosection preparation, fixation, and immunostaining were performed as previously described [76]. Images were acquired using a Leica DMI 6000 microscope with LAS AF software and analyzed and quantified using LAS AF and ImageJ.

The following primary antibodies were used: anti Dystonin, rabbit, Abcam ab234644, 1:200; anti GFP, chicken, Aves Labs GFP‐1020, 1:600; anti MAP1B, rabbit, Abcam ab224115, 1:200; anti NKX6‐1, goat, R&D systems AF5857, 1:300; anti ghrelin (C‐18), goat, Santa Cruz sc‐10368, 1:200; anti GIP (Y‐20), goat, Santa Cruz, sc‐23554, 1:200; anti Sox9, rabbit, Millipore AB5535, 1–300; anti Nkx6‐1, goat, R&D systems AF5857, 1–300. The following secondary antibodies were used: donkey anti‐rabbit IgG 555, Invitrogen A31572, 1:800; donkey anti‐chicken IgY Cy2, Dianova 703‐225‐155, 1:800; donkey anti‐goat, Invitrogen A21082, 1:800.

### 4.7 Protein Translation Assays by O‐Propargyl‐Puromycin (OPP) Labeling

To measure nascent protein synthesis in mouse primary pancreatic cells, E14.5 pancreata were dissected and cultured on Matrigel as described previously [59, 77]. One day after culture, cells were incubated with OPP (50 µg/ml) for 30 min, followed by the Click‐iT Plus OPP Protein Synthesis Assay Kit (Life Technologies, C10456). Samples were then stained with epithelial and endocrine markers and imaged.

For human stem cell‐derived islets, cells were cultured with OPP (50 µg/ml) for 30 min at S5.4 and processed with the same kit. After labelling, cells were stained with synaptophysin (Dako Omnis, M0776) at 4°C overnight. Flow cytometry was performed on a FACS‐Aria III (BD Bioscience), and data were analyzed using FlowJo.

### 4.8 Proteomics Analysis

Samples were lysed in SDC buffer (2% SDC, 100 mM Tris‐HCl, pH 8.5) at 95°C for 10 min with shaking (1000 rpm), followed by sonication in high mode (30 s on/30 s off, 10 cycles; Bioruptor Plus, Diagenode). Protein concentrations were quantified using the BCA assay, and 25 µg of total protein was used for subsequent processing. Reduction was carried out with 10 mM TCEP and alkylation with 40 mM CAA at 45°C for 10 min under shaking (1000 rpm, protected from light). Proteolytic digestion was performed overnight at 37°C with trypsin and LysC at a protease‐to‐protein ratio of 1:40, under constant shaking. Resulting peptides were acidified with 2% TFA in isopropanol (1:1, v/v). Desalting and cleanup were performed using custom‐made StageTips packed with three SDB‐RPS layers (3 M Empore). StageTips were conditioned sequentially with 100% acetonitrile, 1% TFA in 30% methanol, and 0.2% TFA. Acidified peptide mixtures were loaded, passed through, and washed with 1% TFA in ethyl acetate, 1% TFA in isopropanol, and 0.2% TFA. Elution was performed with 60 µL of elution buffer (80% ACN, 1.25% NH_4_OH). Eluted peptides were dried by vacuum centrifugation (40 min, 45°C) and reconstituted in 6 µL of loading buffer (2% ACN, 0.1% TFA). Final peptide concentrations were assessed by UV absorbance at 280 nm (Nanodrop 2000, Thermo Scientific). A total of 0.5 ug peptide was subjected to LC‐MS/MS analysis. Samples were analyzed on an EASY‐nLC 1200 system (Thermo Fisher Scientific) coupled to an Orbitrap Exploris 480 mass spectrometer (Thermo Fisher Scientific) with a nano‐electrospray ion source. The instrument was operated with a FAIMS Pro interface using two compensation voltages (−50 V and –70 V). Peptide separation was achieved at 60°C on a 50 cm × 75 µm inner diameter column packed in‐house with 1.9 µm C18 Reprosil particles (Dr. Maisch GmbH). Chromatography employed a binary solvent system consisting of buffer A (0.1% formic acid in water) and buffer B (80% acetonitrile, 0.1% formic acid), delivered at 300 nL/min. A 60‐min gradient was applied: 5–20% B over 30 min, 20–29% B over 9 min, 29–45% B over 6 min, and 45–95% B over 5 min, followed by a 5‐min wash at 95% B and re‐equilibration to 5% B within 5 min. MS data were acquired in DIA mode with a 2 s duty cycle. Full MS scans were recorded across m/z 300–1650 at 120,000 resolution (m/z 200), AGC target of 3 × 10^6^, and custom maximum injection time. Fragmentation of precursor ions was performed by HCD at normalized collision energy 30, and MS/MS scans were acquired at 15,000 resolution (m/z 200), with an AGC target of 1000% and custom maximum injection time. Raw LC‐MS/MS spectra were processed in Spectronaut (version 15.7) using the directDIA workflow against the mouse UniProt FASTA database (UP000000589_10090), including the MaxQuant contaminant database. Label‐free protein quantification was performed with MaxLFQ settings. Unless stated otherwise, default search parameters were applied. Downstream analysis was conducted in Perseus (version 1.6.14.0). Within Perseus, contaminant proteins were excluded, and only proteins with at least two valid values in one experimental condition across biological replicates were retained. Missing values were imputed from a Gaussian distribution (width = 0.3, downshift = 1.8). Differential expression analysis was conducted using a two‐sample Student's t‐test with permutation‐based FDR control (FDR = 0.1, s0 = 0). Significantly regulated proteins were clustered hierarchically using Euclidean distance after z‐score normalization of median abundances. Pathway enrichment analysis was performed using Metascape [78].

### 4.9 Single‐Cell Multiome (snRNA‐seq/snATAC‐seq) Preprocessing

Sequencing libraries were aligned and quantified with Cell Ranger ARC v2.0.2 against the GRCm38 mouse reference (Ensembl release 102). Downstream processing used Scanpy (v1.10.2), Muon (v0.1.3) and Signac (v1.13.0). For each sample, ambient gene‐expression probabilities were estimated with DropletUtils (v1.22.0), and genes exceeding a per‐sample ambient threshold were flagged as ambient.

#### 4.9.1 Quality Control and Filtering

For each sample, low‐quality nuclei were removed per modality using thresholds set from that sample's QC distributions. On the gene‐expression modality, nuclei were filtered on mitochondrial fraction (mt‐/MT‐genes), total UMI counts and number of detected genes; ribosomal fraction (Rps/Rpl, RPS/RPL) was computed for reference. ATAC quality was assessed with Signac and Muon: nucleosome signal was computed from 10^6^ fragments and TSS enrichment from the top 2000 expressed transcription start sites (GRCm38/Ensembl 102 annotation), after which nuclei were filtered on ATAC counts, TSS enrichment and nucleosome signal. Thresholds common to all three retained replicates were a mitochondrial fraction < 0.25, > 1300 detected genes, total counts < 40 000, ATAC counts < 80 000, TSS enrichment between 0.5 and 15, and nucleosome signal < 1.3. Only the lower count bounds were set per replicate: total counts > 1500 (NVF), > 1,250 (FVF replicate 1) and > 2500 (FVF replicate 2), and ATAC counts > 3000 (NVF and FVF replicate 1) and > 2000 (FVF replicate 2). To merge chromatin data across samples, peaks were combined with the reduce function of GenomicRanges (v1.54.1), restricted to standard chromosomes, re‐quantified per sample with Signac, and stored together with gene‐expression data in a merged object.

#### 4.9.2 Doublet Detection and Consensus Scoring

Doublets were called on the gene‐expression modality with Scrublet (v0.2.3), DoubletDetection (v4.2), scDblFinder (v1.11.4), DoubletFinder (v2.0.3) and SOLO (scvi‐tools v0.17.1), and on the ATAC modality with scDblFinder and its AMULET implementation. For each nucleus, a consensus doublet score was defined as the integer number of the seven callers that classified it as a doublet; nuclei with a consensus score greater than three were designated high‐confidence doublets.

#### 4.9.3 Normalization

ATAC counts were TF‐IDF normalized using Signac (log‐TF‐IDF). Gene‐expression counts were normalized with SCTransform (v0.3.3; vst.flavor = “v2”, clip.range = c(−√n, √n) with N the number of cells, all genes returned). The SCT log‐normalized values (sct_logcounts) served as the working expression matrix for visualization and marker analysis.

#### 4.9.4 Integration and Iterative Multiplet Removal

Gene expression and ATAC modalities were integrated with a MultiVI model (scvi‐tools v1.1.2; n_hidden = 1,024, n_latent = 50, n_layers_encoder = n_layers_decoder = 4, deeply_inject_covariates = True), using modality as the batch key and sample as a categorical covariate. On the MultiVI latent space, a k‐nearest‐neighbor graph (n_neighbors = 20, correlation metric) and Leiden clustering (resolution up to 3) were computed, and clusters enriched for consensus doublet calls were removed iteratively until none remained; remaining individual high‐confidence doublets were then excluded.

#### 4.9.5 Clustering and Annotation

Cells were embedded and clustered on the integrated multiome representation (MultiVI latent space). Cells were Leiden‐clustered (resolution 1.25; subclustered at 0.15–0.6 where required) and annotated with canonical markers. Intestinal clusters were annotated with Lgr5, Olfm4, Dmbt1, Arg2, Sis, Dclk1, Sox4, Pou2f3, Muc2, Dll1, Lyz1, Neurog3, Fev, Arx, Pax4, Lmx1a, Reg4, Isl1, Sst, Gcg, Cck, Gip, Ghrl. Pancreatic clusters (Figure S1d) were annotated with markers Sox9, Neurog3, Fev, Arx, Ghrl, Gcg, Pdx1, Ins1, Sst, Hhex.

### 4.10 Gene Regulatory Network Inference With SCENIC^+^

Enhancer‐driven gene regulatory networks were inferred with SCENIC+ (v1.0a1). Per dataset, a cisTopic object was built from the fragment × peak matrix with pycisTopic, excluding ENCODE blacklist regions (hg38‐blacklist.v2 for human; the corresponding mm10 blacklist for mouse). Topic models were trained with MALLET latent Dirichlet allocation, and candidate enhancer regions were derived by binarizing region–topic contributions using both Otsu thresholding and the top 5000 regions per topic. Motif enrichment on candidate enhancers was performed with pycisTarget using the v10nr_clust motif collection (motifs‐v10‐nr; HGNC/MGI annotation), combining cisTarget and DEM analyses. SCENIC+ objects were assembled with create_SCENICPLUS_object (nr_cells_per_metacells = 1). Enhancer‐to‐gene relationships were computed by gradient‐boosting regression, TF‐to‐gene relationships from the species‐specific TF list, and enhancer‐driven regulons (eRegulons) constructed with the GSEA‐based build_grn approach. Standard eRegulon filtering (apply_std_filtering_to_eRegulons) retained high‐confidence activator regulons (≥15 target genes). Per‐cell regulon activity was quantified with AUCell, regulon specificity scores computed per cell type, and TF–cistrome correlations assessed on pseudobulks (5 cells per pseudobulk).

### 4.11 Integration of the Unpaired human Pancreas Dataset for SCENIC^+^

For the human embryonic/fetal pancreas, in which scRNA‐seq (weeks 8–12) and snATAC‐seq (week 12) were profiled in separate assays, the two modalities were integrated and matched at single‐cell resolution using scGLUE (v0.3.2) prior to SCENIC+, so that gene‐expression and chromatin profiles shared a common cell index (nr_cells_per_metacells = 1). All other datasets (mouse pancreas, SC‐islets and mouse intestine) used genuinely paired snRNA/snATAC profiles from the same nuclei.

### 4.12 TF–TF Correlation Networks and Cross‐Dataset Regulon Comparison

Top TF–TF activator relationships (ρ > 0.2) from the SCENIC+ activator matrices were displayed as directed circular networks; node size denotes each TF's summed absolute correlation (Σ|ρ|) and edges denote positive regulatory correlations. Regulon‐activity (AUC) matrices were compared between predefined regulon subsets across species with Mantel tests (Bray–Curtis distances, permutation p; linkET v0.0.7.4).

### 4.13 Differential Expression and Definition of Highly Enriched Genes (MAST)

For each dataset, cells were labelled endocrine‐progenitor (EP) versus non‐EP using established markers (for example, NEUROG3 for EPs; SOX9, MAFB, SST for ductal, β and δ populations), retaining genes expressed in at least 10–15% of cells. Differential expression was performed with MAST using a hurdle model that accounted for cellular detection rate and sample‐specific covariates, returning per‐gene log2 fold change and FDR‐adjusted *p*‐values. Highly enriched genes (HEGs) were defined as EP‐up genes with FDR < 0.05 and log2 fold change ≥ 1.5 (for each dataset, the EPs‐vs‐Others and Fev^+^‐vs‐Others contrasts were computed separately and merged). For visualization, per‐cell‐type mean expression of HEGs was row Z‐scored and clustered with ClusterGVis [CJ1.1](k‐means), and per‐cluster Gene Ontology (Biological Process) enrichment was computed with clusterProfiler and displayed alongside the corresponding heatmap clusters.

### 4.14 Cross‐Species and Cross‐Dataset Comparison

Mouse and human genes were matched to one‐to‐one orthologs using Ensembl BioMart (biomaRt v2.68.0). All cross‐species comparisons (shared transcription factors, shared HEGs, shared TF‐target pairs and shared glycemic‐risk genes) were computed on this ortholog‐harmonized gene space. For each transcription factor present in two or more datasets, the number of common targets (Tables S4a/S7a/S9b) was defined as the intersection of its predicted target lists after ortholog mapping.

### 4.15 NGN3 CUT&RUN Target Assignment

Neurog3 binding sites were derived from CUT&RUN data of embryonic mouse Ngn3^+^ cells (GSE270915) and reprocessed as described in that study, restricted to the E14.5 Ngn3 datasets. In brief, overrepresented sequences were identified with FastQC (v0.11.3) and removed, together with sequencing adaptors and primers, using Trimmomatic (v0.38). Reads were aligned to the Mus musculus genome (mm10) with Bowtie2 (v2.2.5, default parameters); PCR‐duplicate reads were removed with SAMtools (v1.3.1) and only reads with mapping quality ≥ 30 were retained. Peaks were called per sample with MACS2 (v2.2.6) and retained at *p* < 10^−^^3^, and reproducibility between biological replicates was assessed with the IDR framework (v2.0.4.2), retaining Ngn3 peaks at IDR thresholds of 250 (Ngn3low) and 400 (Ngn3high); peaks overlapping ENCODE blacklist regions were discarded. Peaks within 2 kb of a transcription start site were defined as promoter‐associated, and tag intensities were quantified as tags per 10 million (TP10M) with HOMER (v4.7.2). Ngn3 target genes from the CUT&RUN analysis were intersected with the SCENIC^+^‐predicted NGN3 targets shared between mouse and human (after Ensembl BioMart v2.68.0 ortholog mapping); this overlap defined high‐confidence Ngn3 targets supported by both regulatory‐network prediction and direct binding (Figure 2a; Table S4b). For the in vitro comparison (Figure 3f), NGN3 target genes were taken directly from the published CUT&RUN results of stem cell‐derived endocrine cells (GSE179264) without reprocessing raw reads, and intersected with SCENIC^+^ NGN3 targets shared between the human in vivo and in vitro (SC‐islet) datasets (Table S7b).

### 4.16 Gene Ontology and Pathway Activity Scoring

Gene ontology enrichment of targets was performed using Metascape [78], and significantly enriched terms (adjusted P < 0.05) were visualized as bubble plots based on −log_10_(P) values and gene ratios.

### 4.17 Cell‐Cell Communication Analysis Using CellChat

To assess cellular crosstalk between different cell types of different organ and species, intercellular interactions based on ligands and receptors were inferred and visualized by CellChat(v2.1.2) [79]. In brief, gene expression data of cells and assigned cell type were used as input and core functions were run with standard parameters to infer communication networks and signaling pathways. Interaction events were categorized into secretory signals, cell‐cell contact, and extracellular matrix (ECM) receptors based on the molecular characteristics of the ligand‐receptor pair. Special attention was given to the dynamics of interaction changes, such as the gain or loss of interactions among general cell types and the shifts in outgoing and incoming interactions within cell clusters.

### 4.18 RNA Velocity and Pseudotime Trajectory Analysis

RNA velocity analysis was performed to infer dynamic transcriptional progression at single‐cell resolution across four datasets. For each dataset, spliced and unspliced transcript counts were quantified using velocyto (v0.17.17), and the resulting loom files were imported into scVelo [80] within the Scanpy framework (v1.10.2). Cells and genes passing the same quality‐control thresholds used for the scRNA‐seq analysis were retained. Normalized and log‐transformed counts were used to compute first‐ and second‐order moments across the nearest‐neighbor graph (pp.moments, n_neighbors = 30). RNA velocity vectors were then estimated using the dynamical model implemented in scVelo, which captures transcriptional kinetics and cell‐state transitions beyond steady‐state assumptions. Latent time derived from the dynamical model was used as the pseudotime axis for each dataset, providing a continuous ordering of cells along developmental trajectories. The same low‐dimensional UMAP embedding obtained from Seurat/Scanpy integration was used to visualize the inferred trajectories; velocity streams and pseudotime gradients were plotted on the UMAP embedding to illustrate the direction and progression of cell states. For consistent visualization across datasets, the pseudotime ordering obtained from scVelo was exported to Monocle3 (v1.3.7). Within Monocle3, cells were ordered along principal‐graph trajectories using the learn_graph() and order_cells() functions, anchored to lineage‐defining progenitor clusters (for example, endocrine progenitors). This approach enabled consistent visualization of developmental trajectories and lineage bifurcations across species and experimental conditions.

### 4.19 Glycemic Trait Association Analysis of EP‐Enriched Target Genes

HEG–trait associations were retrieved from the Open Targets Platform (https://platform.opentargets.org) [44]. Human gene symbols were mapped to Ensembl identifiers, and mouse intestinal HEGs were first mapped to their human orthologs. Associations were restricted to Experimental Factor Ontology terms for glycated haemoglobin (HbA1c; EFO_0004541) and type 2 diabetes (a curated set of strict T2D terms merged into a single “T2D” category). Genes with an Open Targets association score ≥ 0.30 (score range 0–1) for either trait were considered associated; multiple ontology hits per gene–trait pair were aggregated to the maximum score.

### 4.20 Identification of SC‐EC‐Specific Genes

SC‐EC‐specific genes were defined through a multi‐step filtering strategy. Starting from EC‐specific differentially expressed genes in the SC‐S7 dataset reported by Balboa et al. (accession number: GSE167880), we excluded genes expressed in human or mouse pancreatic in vivo datasets and retained those highly and selectively expressed in intestinal EC cells. To identify putative transcriptional regulators, SC‐EC‐specific genes were intersected with curated TF–target interactions from the SCENIC^+^ activator database, retaining interactions with an importance (TF‐to‐gene) score > 2 for visualization. For this driver inference, SC‐EC‐enriched genes were mapped onto our progenitor‐enriched, paired multiome SC‐islet dataset [38], as the two datasets are complementary: the former maximizes EC representation for gene discovery, the latter progenitor resolution for regulator inference.

### 4.21 In Silico Transcription‐Factor Knockout (CellOracle)

Candidate SC‐EC regulators were tested by in silico knockout with CellOracle [81] (v0.20.0) on the in vitro stem cell‐derived islet multiome dataset (stage 5) [38], as an orthogonal computational corroboration of the experimental silencing results and independently of the SCENIC^+^ regulons used to nominate drivers in Figure 5. A cell‐type‐specific base gene regulatory network was reconstructed within CellOracle from the SC‐islet chromatin‐accessibility and expression data, providing a regulatory model distinct from the SCENIC^+^ networks. Analysis was restricted to the SC‐EC differentiation lineage (Pre‐Endocrine → endocrine progenitors → β‐progenitors → enterochromaffin cells; *n* = 4288 cells). The lineage was defined by pre‐specified criteria, i.e. k‐nearest‐neighbor edge‐weight composition and raw‐count marker expression (Figure S11a,b). Pseudotime was computed with Palantir on the lineage‐restricted dataset (Figure S11c). A reference differentiation vector field was derived from the Palantir pseudotime gradient on a 40 × 40 grid; the mass‐filter threshold was re‐derived on the lineage subset (primary value 20; sensitivity analysis at 54) with a grid‐smoothing neighborhood of N_KNN_GRID = 30 (sensitivity 50 and 80), yielding 237 mass‐passing grid points. For each knockout, TF expression was set to zero, propagated through the GRN and projected onto the reference grid. The perturbation score was defined as the inner product of the simulated shift and reference vectors; negative values indicated displacement against the direction of differentiation. For the negative control, non‐zero GRN coefficients were permuted within each cluster, preserving network density and coefficient distributions (Figure S11d,e). Observed and randomized perturbation scores were compared at matched grid points using two‐sided Wilcoxon signed‐rank tests with Bonferroni correction. A pre‐specified Rayleigh test of the directional coherence of the reference field was applied per pseudotime stratum. Cell‐state transitions were simulated using the CellOracle Markov‐chain framework (Figure S11f,g).

### 4.22 Single‐Cell Spatial Transcriptomic Analysis

Xenium spatial transcriptomics data from the developing human pancreas (8‐17 WPC) were obtained from a recent publication [41]. To visualize gene expression across tissue sections, transcript counts were normalized to a total of 10,000 transcripts per cell and log1p‐transformed using Scanpy [82], before visualizing spatially using Squidpy [83].

Using cell type annotations from the TERRA manuscript, we subsetted the dataset to trunk, ductal, endocrine progenitor, and endocrine cells. Dimensionality reduction was performed using scVI [84], with sample included as a batch covariate, the total number of detected transcripts per cell included as a continuous covariate, and a 10D latent space. A k‐nearest neighborhood graph was computed from the scVI embeddings, and a UMAP was generated using Scanpy [82].

Single‐nuclei RNA‐sequencing data from fetal samples were downloaded from the European Genome‐phenome Archive (EGA) [40] with accession number EGAS50000001872. Seurat (v5) was used to select ductal cells, progenitor cells, alpha cells, delta cells, epsilon cell, and beta cells and generate the UMAP plot. ggplot2 was used to create dotplots.

### 4.23 Statistical Analysis

Data are presented as mean ± SD, with individual biological replicates shown as dots where applicable. Sample sizes (n) are given in the corresponding figure legends. Two‐group comparisons of qPCR and OPP data were assessed using two‐tailed Student's t‐test, with *p* < 0.05 considered significant. Statistical procedures specific to each dataset (proteomics, single‐cell/single‐nucleus, MAST differential expression, SCENIC+ regulon and Mantel‐test comparisons, CellOracle in silico perturbation, and CellChat ligand–receptor inference) are detailed in their respective sections above, including test type, multiple‐testing correction, and significance thresholds. Analyses were performed using GraphPad Prism, Perseus (v1.6.14.0), Scanpy, Squidpy, scVI, Seurat (v5), MAST, SCENIC^+^ (v1.0a1), CellChat, CellOracle, Palantir, and linkET (v0.0.7.4).

### 4.24 Data Availability 

The multiome of intestinal data generated in this study have been deposited in the GEO database and are available under accession number GSE342091. The mass spectrometry proteomics data have been deposited to the ProteomeXchange Consortium via the PRIDE [85] partner repository with the dataset identifier PXD076279.

## Conflicts of Interest

The authors declare no conflicts of interest.

## Supporting information




**Supporting File 1**: advs77459‐sup‐0001‐SuppMat.pdf.

**Supporting File 2**: advs77459‐sup‐0002‐Tables.zip.

## Data Availability

The data that support the findings of this study are available from the corresponding author upon reasonable request.
