# Supplementary material for: Charting Endocrine Progenitors Across Species and Organs
Source: Adv Sci (Weinh). 2026 Aug 30:e22011. Online ahead of print. doi: 10.1002/advs.202522011 (PMC13526535; doi:10.1002/advs.202522011)
Supplement: Supplementary file 1 [file ADVS-9999-e22011-s002.pdf]

Charting Endocrine Progenitors Across Species and Organs

Changying Jing[1,2,3], Michael Sterr[1,2], Özüm Sehnaz Caliskan[2,4], Lama Saber[1,2,5], Nicole Katarina Rogers[1,2,3], Samuel Ogden[6], Kei Kozawa[1,2], Christos Karampelias[1,2], Jessica Jaki[1,2], Aimee Bastidas-Ponce[1,2,7], Falk Farkas[1,2,5], Oliver Czarnecki[1,2,5], Osvaldo Rodrigues Pereira[1,2,3], Perla Cota[1,2,5], Valerie Vandenbempt[8], Diego Balboa[8], Anika Böttcher[1,2], Natalie Krahmer[2,4], Mohammad Lotfollahi[6,9], Julie B Sneddon[10,11,12,13], Heiko Lickert[1,2,5] and Mostafa Bakhti[1,2]

[1]Institute of Diabetes and Regeneration Research, Helmholtz Munich, Neuherberg, Germany.

[2]German Center for Diabetes Research (DZD), Neuherberg, Germany.

[3]Munich Medical Research School (MMRS), Ludwig Maximilian University (LMU), Munich, Germany.

[4]Institute of Diabetes and Obesity, Helmholtz Munich, Neuherberg, Germany.

[5]School of Medicine, Technical University of Munich (TUM), Munich, Germany.

[6]Wellcome Sanger Institute, Wellcome Genome Campus, Hinxton, Cambridge, UK

[7]Instituto de Biotecnología, Universidad Autónoma Nacional de México, Mexico

[8]Stem Cells and Metabolism Research Program, Faculty of Medicine, University of Helsinki, Helsinki, Finland.

[9]Cambridge Stem Cell Institute, University of Cambridge, Cambridge, UK

[10]Department of Cell and Tissue Biology, University of California, San Francisco, San Francisco, CA, USA.

[11]Department of Anatomy, University of California, San Francisco, San Francisco, CA 94143, USA

[12]Diabetes Center, University of California, San Francisco, San Francisco, CA 94143, USA

[13]Eli and Edythe Broad Center of Regeneration Medicine and Stem Cell Research, University of California, San Francisco, San Francisco, CA 94143, USA

Email for correspondence: mostafa.bakhti@helmholtz-munich.de and heiko.lickert@helmholtz-munich.de

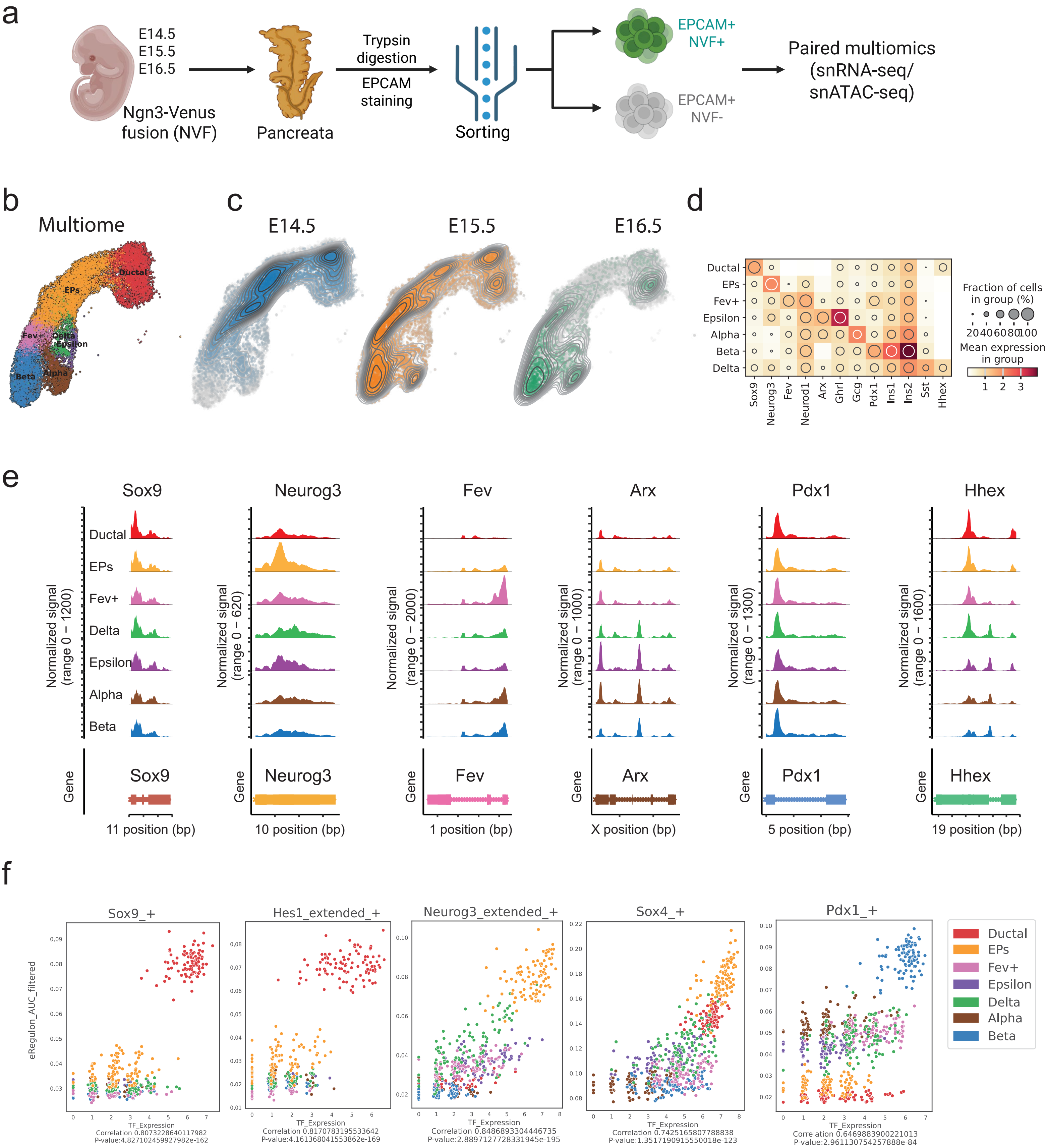
a
E14.5
E15.5
E16.5
Ngn3-Venus fusion (NVF)
Pancreata
Trypsin digestion
EPCAM staining
Sorting
EPCAM+ NVF+
EPCAM+ NVF-
Paired multiomics (snRNA-seq/ snATAC-seq)
b
Multiome
Ductal
EPs
Fev+
Delta
Epsilon
Beta
Alpha
c
E14.5
E15.5
E16.5
d
Ductal
EPs
Fev+
Epsilon
Alpha
Beta
Delta
Sox9
Neurog3
Fev
Neurod1
Arx
Ghrl
Gcg
Pdx1
Ins1
Ins2
Sst
Hhex
Fraction of cells in group (%)
20 40 60 80 100
Mean expression in group
1 2 3
e
Sox9
Neurog3
Fev
Arx
Pdx1
Hhex
Ductal
EPs
Fev+
Delta
Epsilon
Alpha
Beta
Normalized signal (range 0 – 1200)
Normalized signal (range 0 – 620)
Normalized signal (range 0 – 2000)
Normalized signal (range 0 – 1000)
Normalized signal (range 0 – 1300)
Normalized signal (range 0 – 1600)
Gene
11 position (bp)
10 position (bp)
1 position (bp)
X position (bp)
5 position (bp)
19 position (bp)
f
Sox9_+
Hes1_extended_+
Neurog3_extended_+
Sox4_+
Pdx1_+
eRegulon_AUC_filtered
TF_Expression
Correlation 0.8073228640117982
P-value:4.827102459927982e-162
Correlation 0.8170783195533642
P-value:4.161368041553862e-169
Correlation 0.8486893304446735
P-value:2.8897127728331945e-195
Correlation 0.7425165807788838
P-value:1.3517190915550018e-123
Correlation 0.6469883900221013
P-value:2.961130754257888e-84
Ductal
EPs
Fev+
Epsilon
Delta
Alpha
Beta

Figure S1. (a) Schemes show the procedure of performing multiomics of pEP-enriched pancreatic epithelial cells. (b) UMAP plot of mouse embryonic pancreatic epithelial cells, with colors indicating clustering into seven main cell types, including embryonic ductal and endocrine lineages. (c) Density maps showing the distribution of cells at embryonic stages E14.5, E15.5, and E16.5 projected onto the same UMAP space, illustrating the temporal progression of pancreatic lineage differentiation. (d) Dot plot of known cell type-specific gene expression; color intensity represents mean expression in a cluster, and dot size represents the proportion of cells in that cluster expressing the gene. (e) ATAC plots illustrating chromatin accessibility for each cell type across the indicated genomic regions. (f) Plots of specific TFs for each cluster based on both expression and activity.

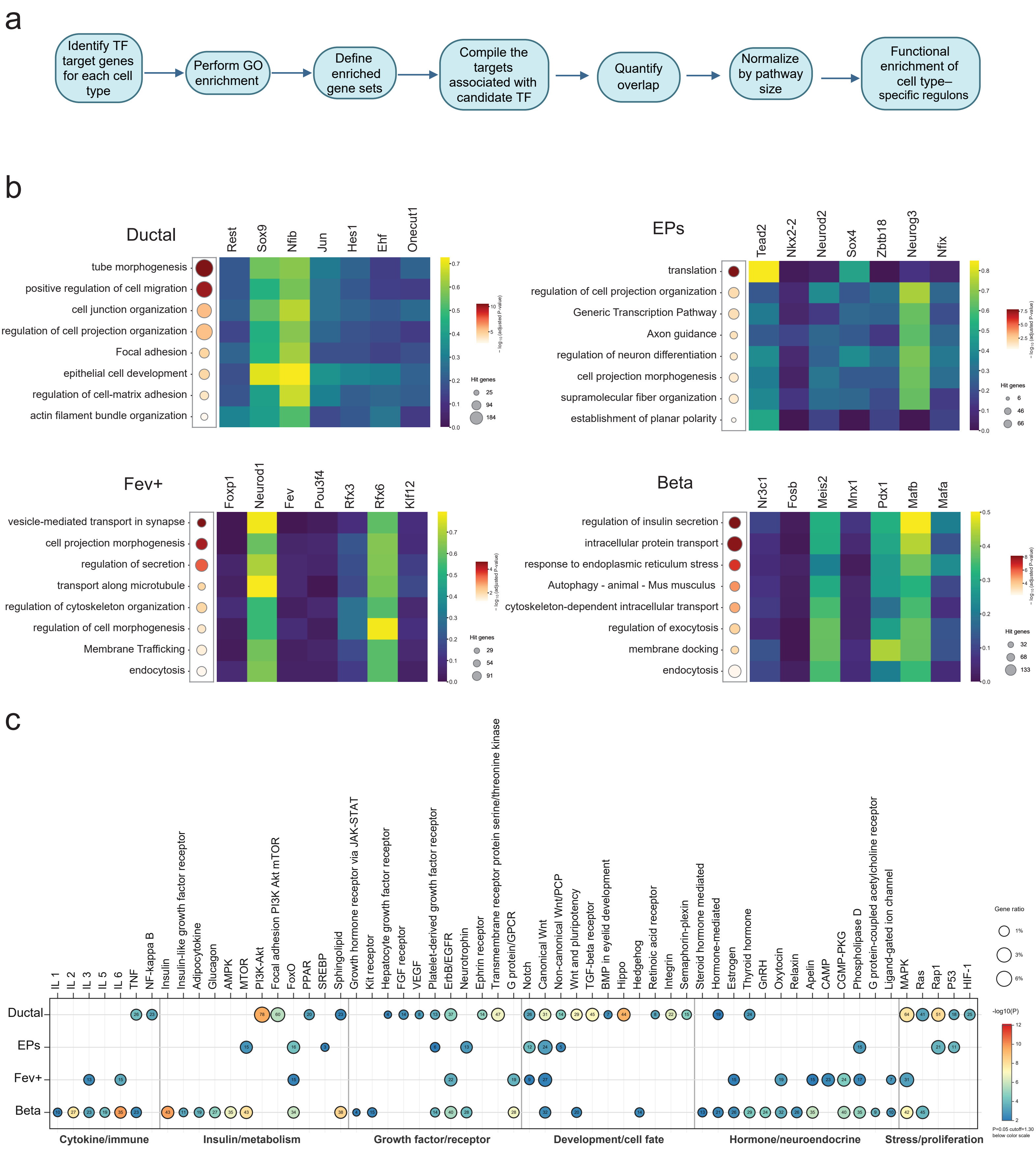
a
Identify TF target genes for each cell type
Perform GO enrichment
Define enriched gene sets
Compile the targets associated with candidate TF
Quantify overlap
Normalize by pathway size
Functional enrichment of cell type–specific regulons
b
Ductal
Rest
Sox9
Nfib
Jun
Hes1
Ehf
Onecut1
tube morphogenesis
positive regulation of cell migration
cell junction organization
regulation of cell projection organization
Focal adhesion
epithelial cell development
regulation of cell-matrix adhesion
actin filament bundle organization
Hit genes
EPs
Tead2
Nkx2-2
Neurod2
Sox4
Zbtb18
Neurog3
Nfix
translation
regulation of cell projection organization
Generic Transcription Pathway
Axon guidance
regulation of neuron differentiation
cell projection morphogenesis
supramolecular fiber organization
establishment of planar polarity
Hit genes
Fev+
Foxp1
Neurod1
Fev
Pou3f4
Rfx3
Rfx6
Klf12
vesicle-mediated transport in synapse
cell projection morphogenesis
regulation of secretion
transport along microtubule
regulation of cytoskeleton organization
regulation of cell morphogenesis
Membrane Trafficking
endocytosis
Hit genes
Beta
Nr3c1
Fosb
Meis2
Mnx1
Pdx1
Mafb
Mafa
regulation of insulin secretion
intracellular protein transport
response to endoplasmic reticulum stress
Autophagy - animal - Mus musculus
cytoskeleton-dependent intracellular transport
regulation of exocytosis
membrane docking
endocytosis
Hit genes
c
IL 1
IL 2
IL 3
IL 5
IL 6
TNF
NF-kappa B
Insulin
Insulin-like growth factor receptor
Adipocytokine
Glucagon
AMPK
MTOR
PI3K-Akt
Focal adhesion PI3K Akt mTOR
FoxO
PPAR
SREBP
Sphingolipid
Growth hormone receptor via JAK-STAT
Kit receptor
Hepatocyte growth factor receptor
FGF receptor
VEGF
Platelet-derived growth factor receptor
ErbB/EGFR
Neurotrophin
Ephrin receptor
Transmembrane receptor protein serine/threonine kinase
G protein/GPCR
Notch
Canonical Wnt
Non-canonical Wnt/PCP
Wnt and pluripotency
TGF-beta receptor
BMP in eyelid development
Hippo
Hedgehog
Retinoic acid receptor
Integrin
Semaphorin-plexin
Steroid hormone mediated
Hormone-mediated
Estrogen
Thyroid hormone
GnRH
Oxytocin
Relaxin
Apelin
CAMP
CGMP-PKG
Phospholipase D
G protein-coupled acetylcholine receptor
Ligand-gated ion channel
MAPK
Ras
Rap1
P53
HIF-1
Ductal
EPs
Fev+
Beta
Gene ratio
1%
3%
6%
-log10(P)
Cytokine/immune
Insulin/metabolism
Growth factor/receptor
Development/cell fate
Hormone/neuroendocrine
Stress/proliferation

Figure S2. (a) Scheme illustrating how the target genes for each cluster were obtained. (b-e) Pathway enrichment analysis of the four main clusters, based on the targets of specific TFs in each corresponding cell type. The major TFs regulating each term are indicated on the right side of each plot. For each cell type, selected terms are shown. (c) Canonical signaling pathways derived from the enriched GO terms, shown across the four clusters together with their inferred activity. The full pathway enrichment results are provided in Supplementary Table 2b-f.

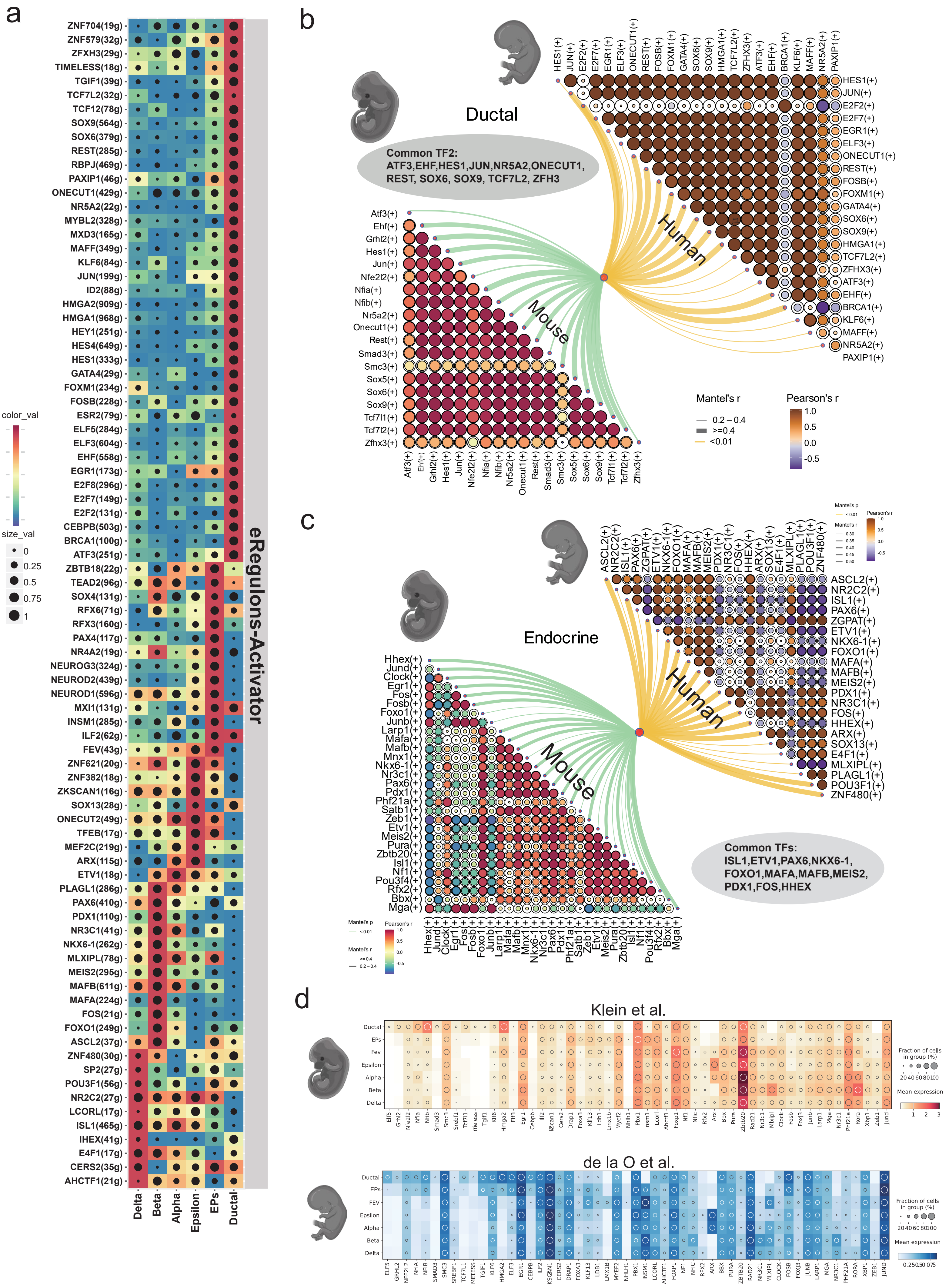
a
ZNF704(19g)
ZNF579(32g)
ZFXH3(29g)
TIMELESS(18g)
TGIF1(39g)
TCF7L2(32g)
TCF12(78g)
SOX9(564g)
SOX6(379g)
REST(285g)
RBPJ(469g)
PAXIP1(46g)
ONECUT1(429g)
NR5A2(22g)
MYBL2(328g)
MXD3(165g)
MAFF(349g)
KLF6(84g)
JUN(199g)
ID2(88g)
HMGA2(909g)
HMGA1(968g)
HEY1(251g)
HES4(649g)
HES1(333g)
GATA4(29g)
FOXM1(234g)
FOSB(228g)
ESR2(79g)
ELF5(284g)
ELF3(604g)
EHF(558g)
EGR1(173g)
E2F8(296g)
E2F7(149g)
E2F2(131g)
CEBPB(503g)
BRCA1(100g)
ATF3(251g)
ZBTB18(22g)
TEAD2(96g)
SOX4(131g)
RFX6(71g)
RFX3(160g)
PAX4(117g)
NR4A2(19g)
NEUROG3(324g)
NEUROD2(439g)
NEUROD1(596g)
MXI1(131g)
INSM1(285g)
ILF2(62g)
FEV(43g)
ZNF621(20g)
ZNF382(18g)
ZKSCAN1(16g)
SOX13(28g)
ONECUT2(49g)
TFEB(17g)
MEF2C(219g)
ARX(115g)
ETV1(18g)
PLAGL1(286g)
PAX6(410g)
PDX1(110g)
NR3C1(41g)
NKX6-1(262g)
MLXIPL(78g)
MEIS2(295g)
MAFB(611g)
MAFA(224g)
FOS(21g)
FOXO1(249g)
ASCL2(37g)
ZNF480(30g)
SP2(27g)
POU3F1(56g)
NR2C2(27g)
LCORL(17g)
ISL1(465g)
IHEX(41g)
E4F1(17g)
CERS2(35g)
AHCTF1(21g)
Delta
Beta
Alpha
Epsilon
EPs
Ductal
eRegulons-Activator
color_val
size_val
0
0.25
0.5
0.75
1
b
Ductal
Common TF2:
ATF3,EHF,HES1,JUN,NR5A2,ONECUT1,
REST, SOX6, SOX9, TCF7L2, ZFH3
Human
Mouse
Mantel's r
0.2 – 0.4
>=0.4
<0.01
Pearson's r
1.0
0.5
0.0
-0.5
c
Endocrine
Human
Mouse
Common TFs:
ISL1,ETV1,PAX6,NKX6-1,
FOXO1,MAFA,MAFB,MEIS2,
PDX1,FOS,HHEX
d
Klein et al.
de la O et al.
Ductal
EPs
Fev
Epsilon
Alpha
Beta
Delta
Fraction of cells in group (%)
20 40 60 80 100
Mean expression

Figure S3. (a) Heatmap plot of activating TFs identified in human embryonic pancreatic lineages. Colors indicate expression levels and dark circles denote activity. (b) The triangular heatmaps comparing ductal-specific TFs between mouse and human, with common TFs highlighted. (c) The triangular heatmaps comparing endocrine-specific TFs between mouse and human, highlighting common TFs. (d) Dot plots showing similar expression patterns of TFs that were not identified as common in the SCENIC^{+} analysis between mouse and human.

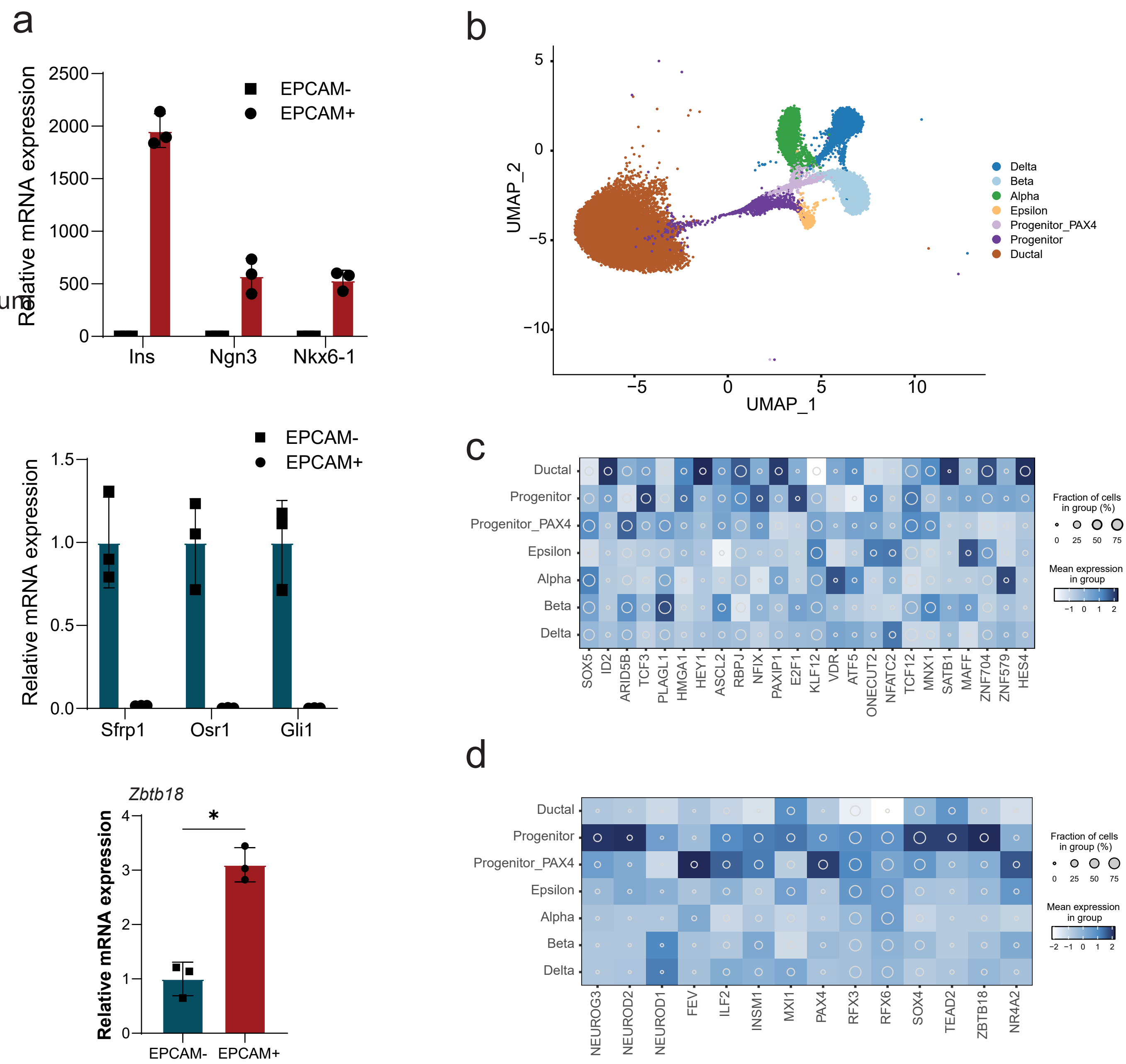

a
Relative mRNA expression
2500
2000
1500
1000
500
0
EPCAM-
EPCAM+
Ins
Ngn3
Nkx6-1
1.5
1.0
0.5
0.0
Sfrp1
Osr1
Gli1
Zbtb18
*
4
3
2
1
0
EPCAM-
EPCAM+
b
UMAP_2
UMAP_1
5
0
−5
−10
10
Delta
Beta
Alpha
Epsilon
Progenitor_PAX4
Progenitor
Ductal
c
Ductal
Progenitor
Progenitor_PAX4
Epsilon
Alpha
Beta
Delta
SOX5
ID2
ARID5B
TCF3
PLAGL1
HMGA1
HEY1
ASCL2
RBPJ
NFIX
PAXIP1
E2F1
KLF12
VDR
ATF5
ONECUT2
NFATC2
TCF12
MNX1
SATB1
MAFF
ZNF704
ZNF579
HES4
Fraction of cells in group (%)
0 25 50 75
Mean expression in group
−1 0 1 2
d
Ductal
Progenitor
Progenitor_PAX4
Epsilon
Alpha
Beta
Delta
NEUROG3
NEUROD2
NEUROD1
FEV
ILF2
INSM1
MXI1
PAX4
RFX3
RFX6
SOX4
TEAD2
ZBTB18
NR4A2
Fraction of cells in group (%)
0 25 50 75
Mean expression in group
−2 −1 0 1 2

Figure S4. (a) qPCR validation of FACS-sorted epithelial and non-epithelial fractions from E15.5 mouse pancreata. Epithelial markers *Ins*, *Ngn3*, and *Nkx6-1* are enriched in the EPCAM+ fraction, while mesenchymal/non-epithelial markers *Sfrp1*, *Osr1*, and *Gli1* are enriched in the EPCAM− fraction. *Zbtb18* is significantly enriched in the EPCAM+ fraction. n = 3; Data are mean ± SD; two-tailed t-test; *P < 0.05. (b) UMAP of a previously generated single-cell dataset of the developing human pancreas, colored by cell-type cluster. (c) Dot plot of species-specific and divergent TFs across the clusters, recapitulating the expression patterns from our human analysis. (d) Dot plot of canonical EP/progenitor-specific TFs, confirming their enrichment in the progenitor clusters. Dot size represents the fraction of cells in the group; color represents mean expression in group.

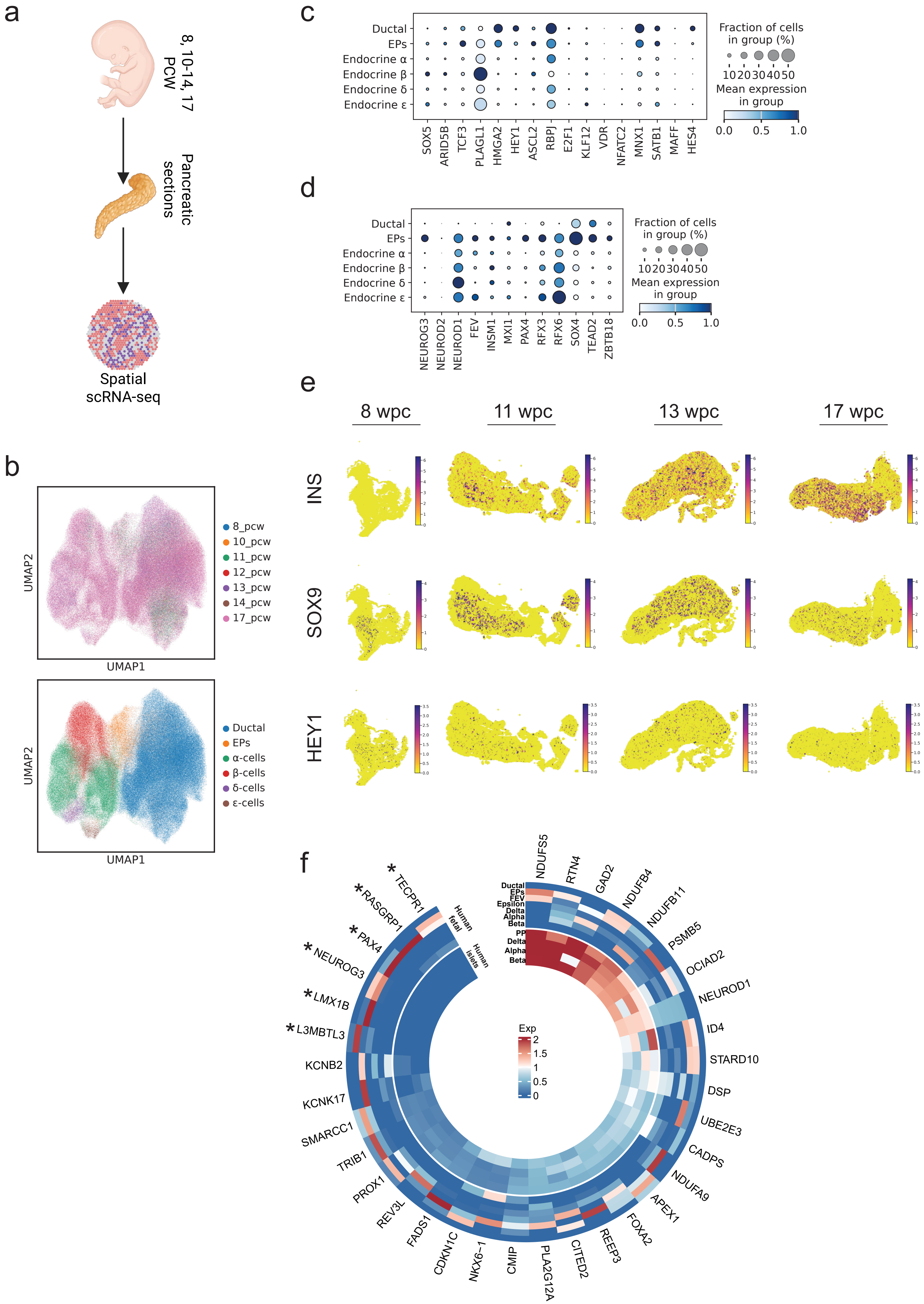
a
8, 10-14, 17 PCW
Pancreatic sections
Spatial scRNA-seq
b
UMAP2
UMAP1
8_pcw
10_pcw
11_pcw
12_pcw
13_pcw
14_pcw
17_pcw
Ductal
EPs
α-cells
β-cells
δ-cells
ε-cells
c
Ductal
EPs
Endocrine α
Endocrine β
Endocrine δ
Endocrine ε
SOX5
ARID5B
TCF3
PLAGL1
HMGA2
HEY1
ASCL2
RBPJ
E2F1
KLF12
VDR
NFATC2
MNX1
SATB1
MAFF
HES4
Fraction of cells in group (%)
10 20 30 40 50
Mean expression in group
0.0 0.5 1.0
d
NEUROG3
NEUROD2
NEUROD1
FEV
INSM1
MXI1
PAX4
RFX3
RFX6
SOX4
TEAD2
ZBTB18
e
8 wpc
11 wpc
13 wpc
17 wpc
INS
SOX9
HEY1
f
Human fetal
Human islets
Ductal
EPs
FEV
Epsilon
Delta
Alpha
Beta
PP
Exp
2
1.5
1
0.5
0
*TECPR1
*RASGRP1
*PAX4
*NEUROG3
*LMX1B
*L3MBTL3
KCNB2
KCNK17
SMARCC1
TRIB1
PROX1
REV3L
FADS1
CDKN1C
NKX6−1
CMIP
PLA2G12A
CITED2
REEP3
FOXA2
APEX1
NDUFA9
CADPS
UBE2E3
DSP
STARD10
ID4
NEUROD1
OCIAD2
PSMB5
NDUFB11
NDUFB4
GAD2
RTN4
NDUFS5

Figure S5. (a) Schematic of the human spatial transcriptomics workflow. Pancreatic sections from developing human pancreas spanning 8, 10-14, and 17 PCW were processed for spatial scRNA-seq. (b) UMAP plots of the human spatial dataset, colored by developmental stage (top) and by cell-type cluster (bottom). (c) Dot plot of species-specific/divergent TFs across ductal, EP, and endocrine clusters, recapitulating most TF expression patterns from the human analysis. (d) Dot plot of canonical EP-specific TFs confirming enrichment in the EP cluster. Dot size represents the fraction of cells in the group; color represents mean expression in group. (e) Spatial expression of *INS*, *SOX9*, and *HEY1* in the developing human pancreas. (f) Circular plot illustrating the expression of selected human pEP HEGs associated with HbA1c and T2D across human embryonic pancreatic lineages and adult islet cells. Asterisks indicate genes that show very low expression in human adult islets.

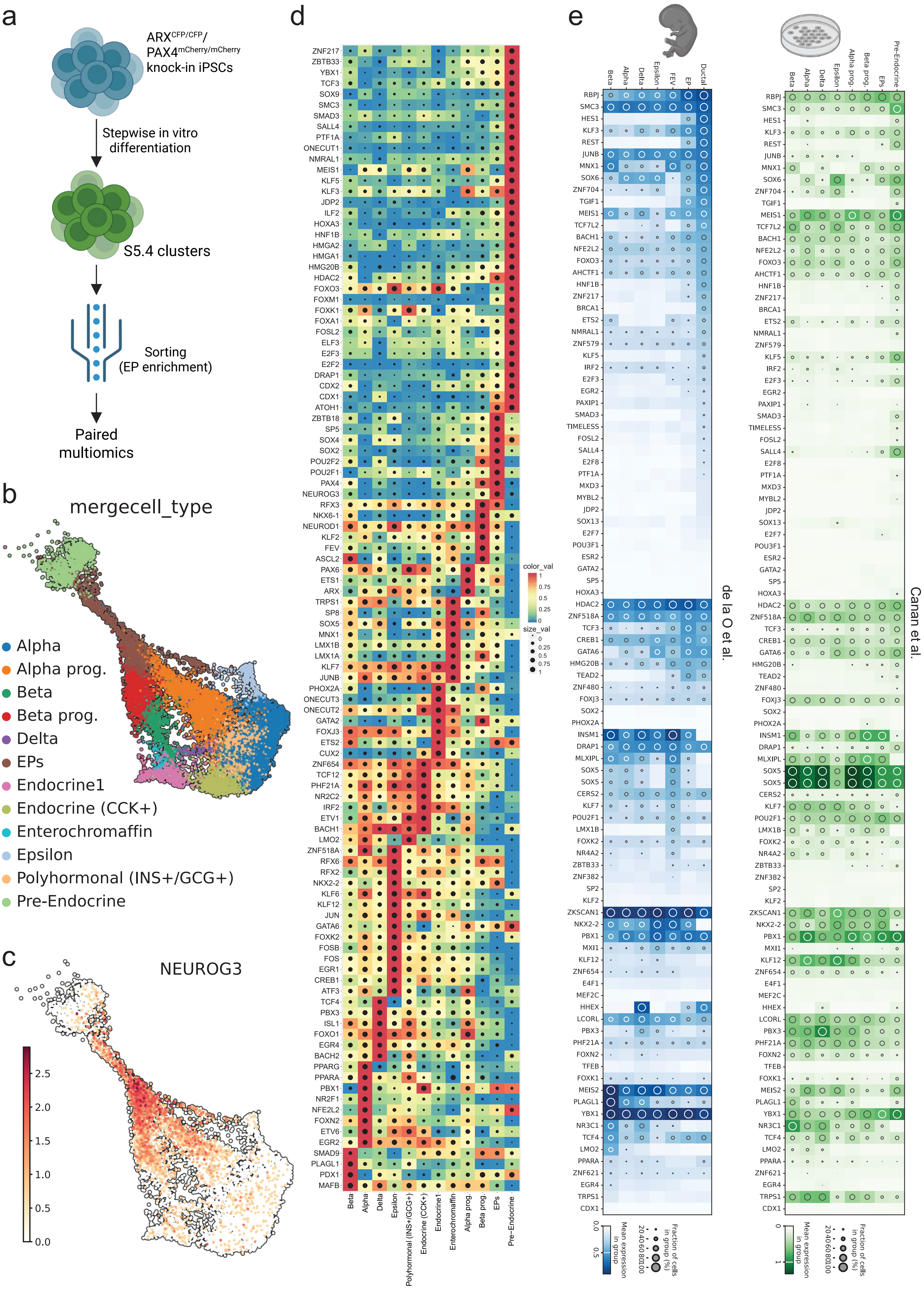
a
ARX CFP/CFP / PAX4 mCherry/mCherry knock-in iPSCs
Stepwise in vitro differentiation
S5.4 clusters
Sorting (EP enrichment)
Paired multiomics
b
mergecell_type
Alpha
Alpha prog.
Beta
Beta prog.
Delta
EPs
Endocrine1
Endocrine (CCK+)
Enterochromaffin
Epsilon
Polyhormonal (INS+/GCG+)
Pre-Endocrine
c
NEUROG3
2.5
2.0
1.5
1.0
0.5
0.0
d
ZNF217 ZBTB33 YBX1 TCF3 SOX9 SMC3 SMAD3 SALL4 PTF1A ONECUT1 NMRAL1 MEIS1 KLF5 KLF3 JDP2 ILF2 HOXA3 HNF1B HMGA2 HMGA1 HMG20B HDAC2 FOXO3 FOXM1 FOXK1 FOXA1 FOSL2 ELF3 E2F3 E2F2 DRAP1 CDX2 CDX1 ATOH1 ZBTB18 SP5 SOX4 SOX2 POU2F2 POU2F1 PAX4 NEUROG3 RFX3 NKX6-1 NEUROD1 KLF2 FEV ASCL2 PAX6 ETS1 ARX TRPS1 SP8 SOX5 MNX1 LMX1B LMX1A KLF7 JUNB PHOX2A ONECUT3 ONECUT2 GATA2 FOXJ3 ETS2 CUX2 ZNF654 TCF12 PHF21A NR2C2 IRF2 ETV1 BACH1 LMO2 ZNF518A RFX6 RFX2 NKX2-2 KLF6 KLF12 JUN GATA6 FOXK2 FOSB FOS EGR1 CREB1 ATF3 TCF4 PBX3 ISL1 FOXO1 EGR4 BACH2 PPARG PPARA PBX1 NR2F1 NFE2L2 FOXN2 ETV6 EGR2 SMAD9 PLAGL1 PDX1 MAFB
Beta Alpha Delta Epsilon Polyhormonal (INS+/GCG+) Endocrine (CCK+) Endocrine1 Enterochromaffin Alpha prog. Beta prog. EPs Pre−Endocrine
color_val 1 0.75 0.5 0.25 0
size_val 0 0.25 0.5 0.75 1
e
Beta Alpha Delta Epsilon FEV EP Ductal
RBPJ SMC3 HES1 KLF3 REST JUNB MNX1 SOX6 ZNF704 TGIF1 MEIS1 TCF7L2 BACH1 NFE2L2 FOXO3 AHCTF1 HNF1B ZNF217 BRCA1 ETS2 NMRAL1 ZNF579 KLF5 IRF2 E2F3 EGR2 PAXIP1 SMAD3 TIMELESS FOSL2 SALL4 E2F8 PTF1A MXD3 MYBL2 JDP2 SOX13 E2F7 POU3F1 ESR2 GATA2 SP5 HOXA3 HDAC2 ZNF518A TCF3 CREB1 GATA6 HMG20B TEAD2 ZNF480 FOXJ3 SOX2 PHOX2A INSM1 DRAP1 MLXIPL SOX5 SOX5 CERS2 KLF7 POU2F1 LMX1B FOXK2 NR4A2 ZBTB33 ZNF382 SP2 KLF2 ZKSCAN1 NKX2-2 PBX1 MXI1 KLF12 ZNF654 E4F1 MEF2C HHEX LCORL PBX3 PHF21A FOXN2 TFEB FOXK1 MEIS2 PLAGL1 YBX1 NR3C1 TCF4 LMO2 PPARA ZNF621 EGR4 TRPS1 CDX1
de la O et al.
0.0 0.5 Mean expression in group
Fraction of cells in group (%) 20 40 60 80 100
Beta Alpha Delta Epsilon Alpha prog. Beta prog. EPs Pre-Endocrine
RBPJ SMC3 HES1 KLF3 REST JUNB MNX1 SOX6 ZNF704 TGIF1 MEIS1 TCF7L2 BACH1 NFE2L2 FOXO3 AHCTF1 HNF1B ZNF217 BRCA1 ETS2 NMRAL1 ZNF579 KLF5 IRF2 E2F3 EGR2 PAXIP1 SMAD3 TIMELESS FOSL2 SALL4 E2F8 PTF1A MXD3 MYBL2 JDP2 SOX13 E2F7 POU3F1 ESR2 GATA2 SP5 HOXA3 HDAC2 ZNF518A TCF3 CREB1 GATA6 HMG20B TEAD2 ZNF480 FOXJ3 SOX2 PHOX2A INSM1 DRAP1 MLXIPL SOX5 SOX5 CERS2 KLF7 POU2F1 LMX1B FOXK2 NR4A2 ZBTB33 ZNF382 SP2 KLF2 ZKSCAN1 NKX2-2 PBX1 MXI1 KLF12 ZNF654 E4F1 MEF2C HHEX LCORL PBX3 PHF21A FOXN2 TFEB FOXK1 MEIS2 PLAGL1 YBX1 NR3C1 TCF4 LMO2 PPARA ZNF621 EGR4 TRPS1 CDX1
Canan et al.
0 1 Mean expression in group
Fraction of cells in group (%) 20 40 60 80 100

Figure S6. (a) Schematic of the *in vitro* differentiation and sorting strategy of the $ARX^{CFP/CFP}/PAX4^{mCherry/mCherry}$ reporter knock-in iPSCs. (b) UMAP plot of SC-islet cells colored by cell type. (c) UMAP plot showing NEUROG3 expression, marking the endocrine progenitor population. (d) Heatmap plot of activating TFs identified in stem cell-derived pancreatic lineages *in vitro*; colors indicate expression levels and dark circles denote activity. (e) Dot plots illustrating similar expression patterns of TFs that were not identified as common in the $SCENIC^+$ analysis between human *in vivo* and *in vitro* systems.

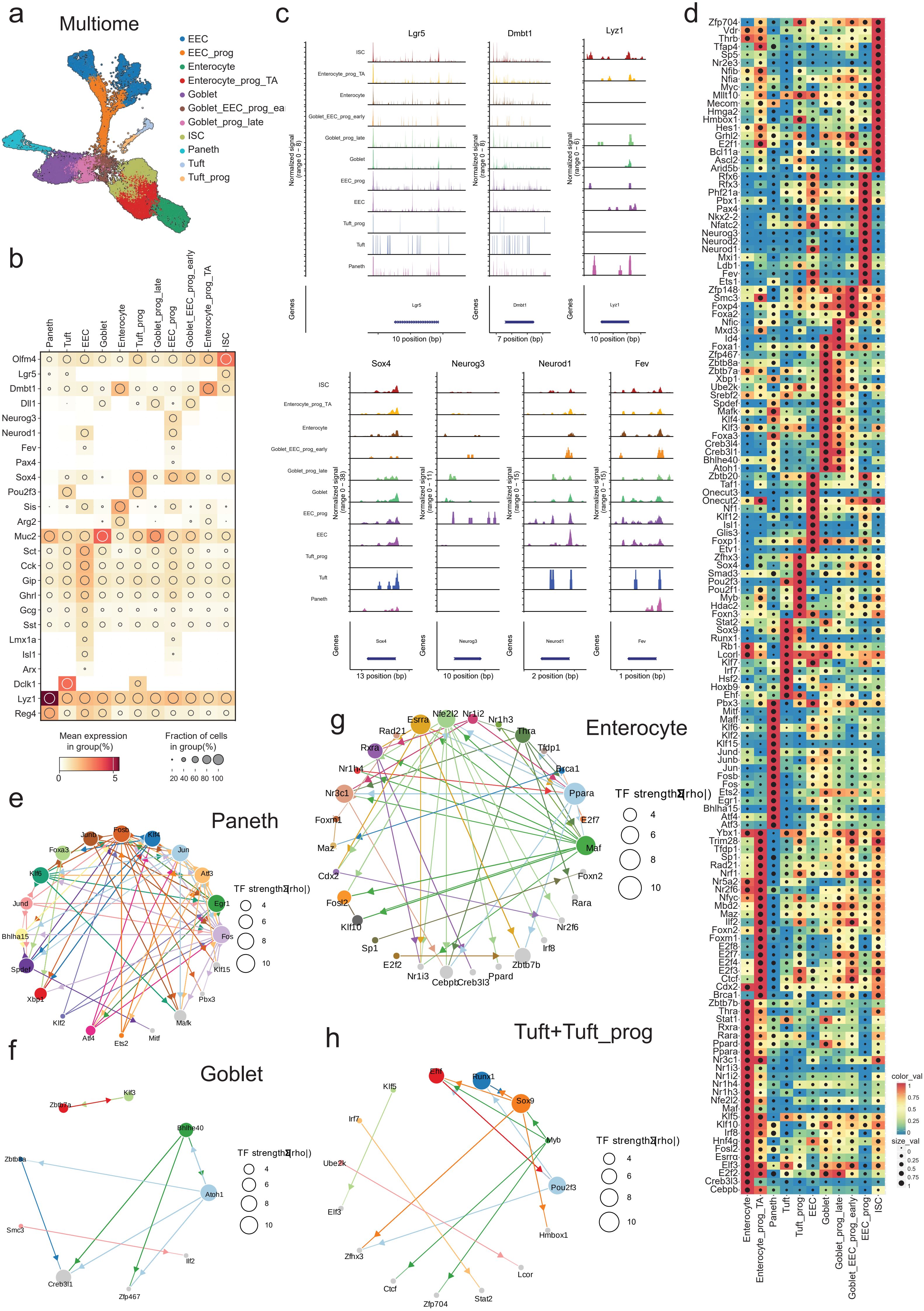

a
Multiome
EEC
EEC_prog
Enterocyte
Enterocyte_prog_TA
Goblet
Goblet_EEC_prog_ea
Goblet_prog_late
ISC
Paneth
Tuft
Tuft_prog
b
Paneth
Tuft
EEC
Goblet
Enterocyte
Tuft_prog
Goblet_prog_late
EEC_prog
Goblet_EEC_prog_early
Enterocyte_prog_TA
ISC
Olfm4
Lgr5
Dmbt1
Dll1
Neurog3
Neurod1
Fev
Pax4
Sox4
Pou2f3
Sis
Arg2
Muc2
Sct
Cck
Gip
Ghrl
Gcg
Sst
Lmx1a
Isl1
Arx
Dclk1
Lyz1
Reg4
Mean expression in group(%)
0
5
Fraction of cells in group(%)
20 40 60 80 100
c
Lgr5
Dmbt1
Lyz1
Sox4
Neurog3
Neurod1
Fev
Genes
e
Paneth
f
Goblet
g
Enterocyte
h
Tuft+Tuft_prog
TF strength∑|rho|)
4
6
8
10
d
color_val
size_val

Figure S7. (a) UMAP plot of 20727 profiled single cells from mouse intestinal epithelium, with colors indicating clustering into 11 main cell types. (b) Dot plot of known cell type-specific gene expression. Color intensity represents mean expression in a cluster, and dot size represents the proportion of cells in that cluster expressing the gene. (c) ATAC plots illustrating chromatin accessibility for each cell type across the indicated genomic regions. (d) Heatmap plot of activating TFs identified in mouse intestinal epithelial lineages; colors indicate expression levels and dark circles denote activity. (e-h) Circular TF correlation networks showing GRNs of Tuft, Enterocyte, Paneth, and Goblet lineages, with arrows indicating downstream targets.

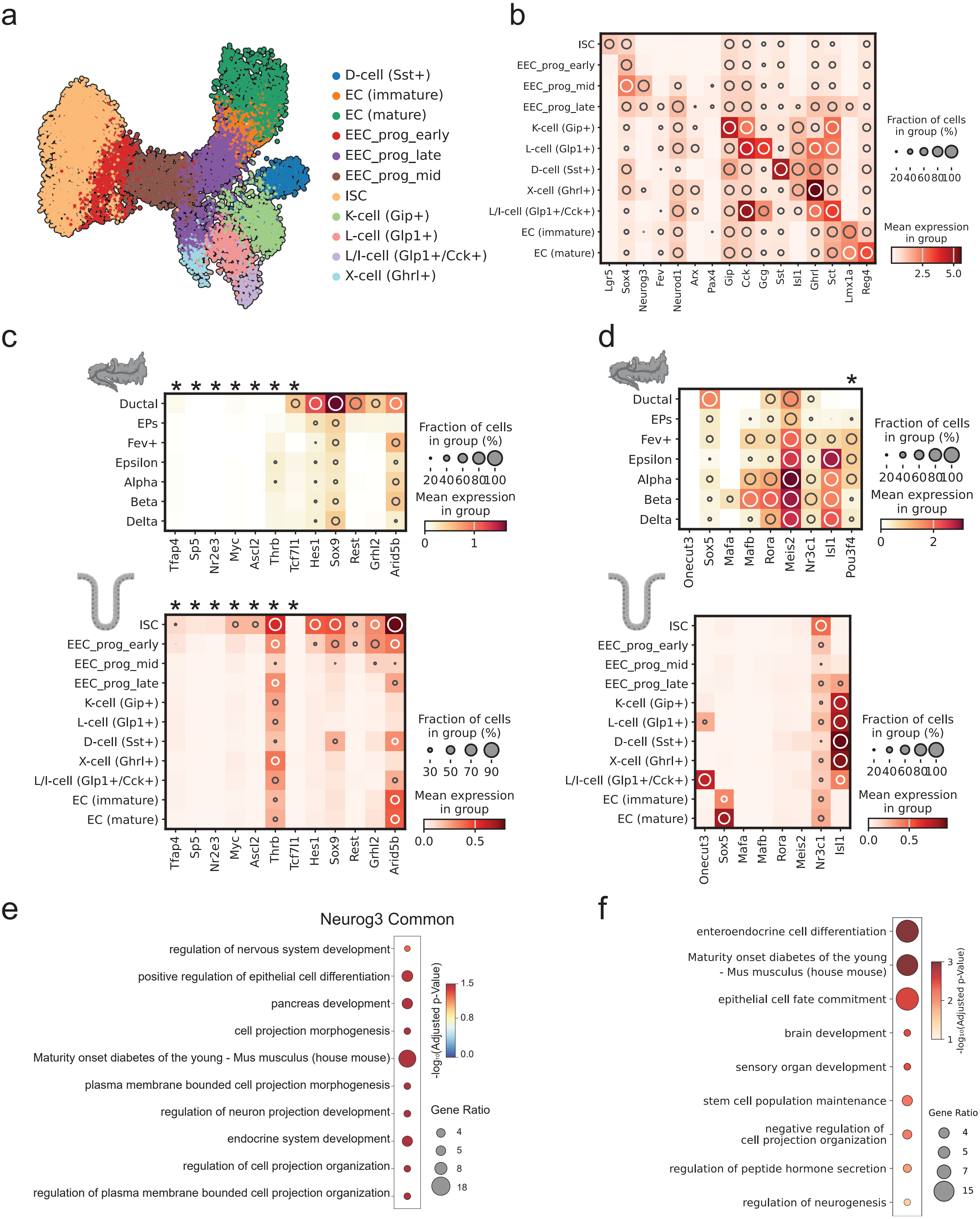
a
D-cell (Sst+)
EC (immature)
EC (mature)
EEC_prog_early
EEC_prog_late
EEC_prog_mid
ISC
K-cell (Gip+)
L-cell (Glp1+)
L/I-cell (Glp1+/Cck+)
X-cell (Ghrl+)
b
ISC
EEC_prog_early
EEC_prog_mid
EEC_prog_late
K-cell (Gip+)
L-cell (Glp1+)
D-cell (Sst+)
X-cell (Ghrl+)
L/I-cell (Glp1+/Cck+)
EC (immature)
EC (mature)
Lgr5 Sox4 Neurog3 Fev Neurod1 Arx Pax4 Gip Cck Gcg Sst Isl1 Ghrl Sct Lmx1a Reg4
Fraction of cells in group (%)
20 40 60 80 100
Mean expression in group
2.5 5.0
c
Ductal
EPs
Fev+
Epsilon
Alpha
Beta
Delta
Tfap4 Sp5 Nr2e3 Myc Ascl2 Thrb Tcf7l1 Hes1 Sox9 Rest Grhl2 Arid5b
Fraction of cells in group (%)
20 40 60 80 100
Mean expression in group
0 1
ISC
EEC_prog_early
EEC_prog_mid
EEC_prog_late
K-cell (Gip+)
L-cell (Glp1+)
D-cell (Sst+)
X-cell (Ghrl+)
L/I-cell (Glp1+/Cck+)
EC (immature)
EC (mature)
Tfap4 Sp5 Nr2e3 Myc Ascl2 Thrb Tcf7l1 Hes1 Sox9 Rest Grhl2 Arid5b
Fraction of cells in group (%)
30 50 70 90
Mean expression in group
0.0 0.5
d
Ductal
EPs
Fev+
Epsilon
Alpha
Beta
Delta
Onecut3 Sox5 Mafa Mafb Rora Meis2 Nr3c1 Isl1 Pou3f4
Fraction of cells in group (%)
20 40 60 80 100
Mean expression in group
0 2
ISC
EEC_prog_early
EEC_prog_mid
EEC_prog_late
K-cell (Gip+)
L-cell (Glp1+)
D-cell (Sst+)
X-cell (Ghrl+)
L/I-cell (Glp1+/Cck+)
EC (immature)
EC (mature)
Onecut3 Sox5 Mafa Mafb Rora Meis2 Nr3c1 Isl1
Fraction of cells in group (%)
20 40 60 80 100
Mean expression in group
0.0 0.5
e
Neurog3 Common
regulation of nervous system development
positive regulation of epithelial cell differentiation
pancreas development
cell projection morphogenesis
Maturity onset diabetes of the young - Mus musculus (house mouse)
plasma membrane bounded cell projection morphogenesis
regulation of neuron projection development
endocrine system development
regulation of cell projection organization
regulation of plasma membrane bounded cell projection organization
-log₁₀(Adjusted p-Value)
1.5
0.8
0.0
Gene Ratio
4
5
8
18
f
enteroendocrine cell differentiation
Maturity onset diabetes of the young - Mus musculus (house mouse)
epithelial cell fate commitment
brain development
sensory organ development
stem cell population maintenance
negative regulation of cell projection organization
regulation of peptide hormone secretion
regulation of neurogenesis
-log₁₀(Adjusted p-Value)
3
2
1
Gene Ratio
4
5
7
15

Figure S8. (a) UMAP plot of mouse intestinal epithelial cells. (b) Dot plot of known cell type-specific gene expression; color intensity represents mean expression in a cluster, and dot size represents the proportion of cells in that cluster expressing the gene. (c) Dot plots illustrating similar expression patterns of TFs that were not identified as common in the SCENIC$^+$ analysis between mouse pancreatic (ductal) and intestinal (ISC) cells. Asterisks indicate genes that are expressed differently in the other organ. (d) Dot plots illustrating similar expression patterns of TFs that were not identified as common in the SCENIC$^+$ analysis between mouse pancreatic (endocrine) and intestinal (EECs) cells. Asterisk indicates gene that were not detected in the other species. (e) Pathway enrichment analysis of the 56 common NGN3 targets shared between mouse and human pancreas and mouse intestine. Selected terms are shown. The list of all terms is provided in Supplementary Table 9e. (f) Pathway enrichment analysis of common HEGs between mouse pEPs and iEPs. Selected terms are shown. The list of all terms is provided in Supplementary Table 9i.

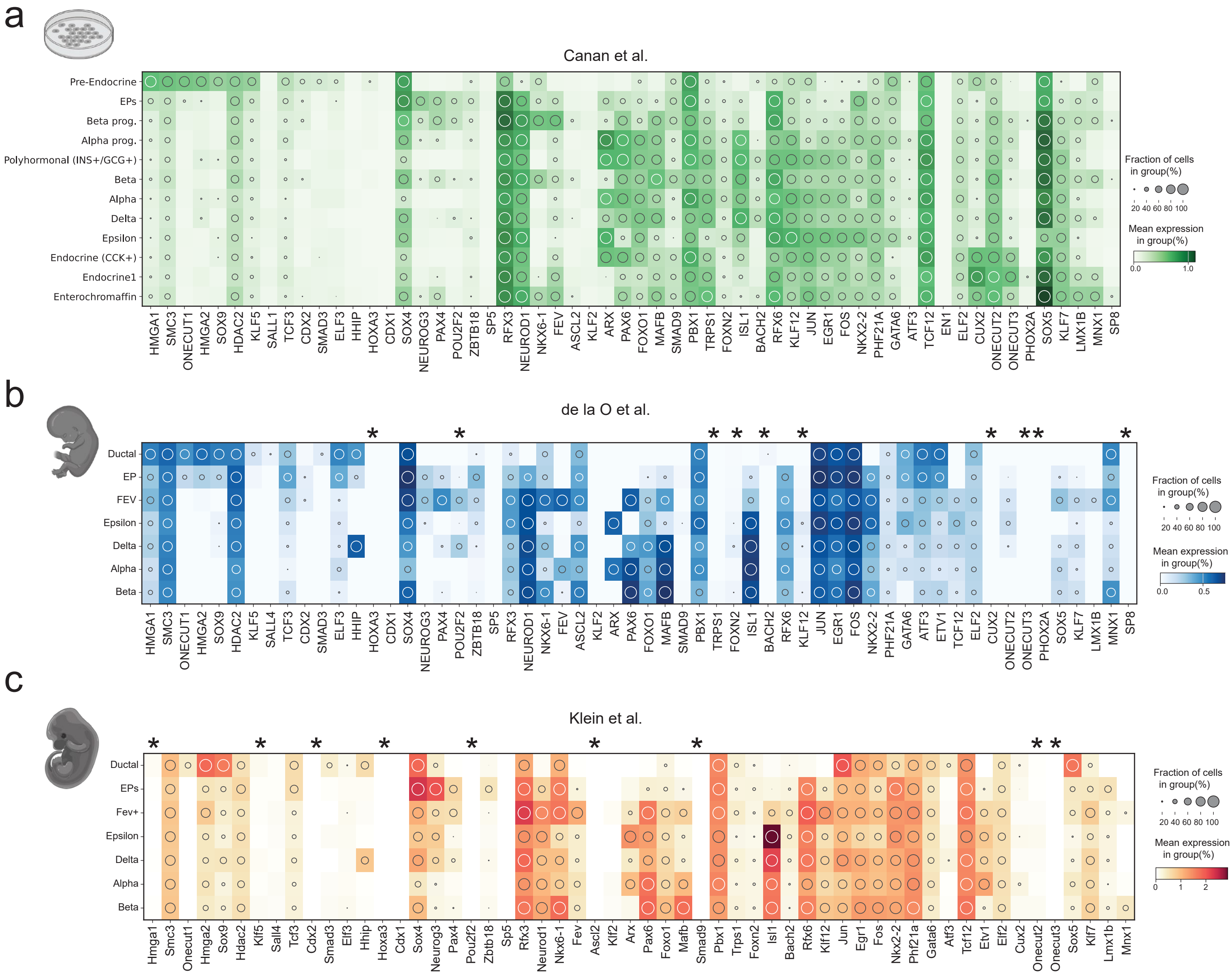
a
Canan et al.
Pre-Endocrine
EPs
Beta prog.
Alpha prog.
Polyhormonal (INS+/GCG+)
Beta
Alpha
Delta
Epsilon
Endocrine (CCK+)
Endocrine1
Enterochromaffin
HMGA1 SMC3 ONECUT1 HMGA2 SOX9 HDAC2 KLF5 SALL1 TCF3 CDX2 SMAD3 ELF3 HHIP HOXA3 CDX1 SOX4 NEUROG3 PAX4 POU2F2 ZBTB18 SP5 RFX3 NEUROD1 NKX6-1 FEV ASCL2 KLF2 ARX PAX6 FOXO1 MAFB SMAD9 PBX1 TRPS1 FOXN2 ISL1 BACH2 RFX6 KLF12 JUN EGR1 FOS NKX2-2 PHF21A GATA6 ATF3 TCF12 EN1 ELF2 CUX2 ONECUT2 ONECUT3 PHOX2A SOX5 KLF7 LMX1B MNX1 SP8
Fraction of cells in group(%)
20 40 60 80 100
Mean expression in group(%)
0.0
1.0
b
de la O et al.
Ductal
EP
FEV
Epsilon
Delta
Alpha
Beta
HMGA1 SMC3 ONECUT1 HMGA2 SOX9 HDAC2 KLF5 SALL4 TCF3 CDX2 SMAD3 ELF3 HHIP HOXA3 CDX1 SOX4 NEUROG3 PAX4 POU2F2 ZBTB18 SP5 RFX3 NEUROD1 NKX6-1 FEV ASCL2 KLF2 ARX PAX6 FOXO1 MAFB SMAD9 PBX1 TRPS1 FOXN2 ISL1 BACH2 RFX6 KLF12 JUN EGR1 FOS NKX2-2 PHF21A GATA6 ATF3 ETV1 TCF12 ELF2 CUX2 ONECUT2 ONECUT3 PHOX2A SOX5 KLF7 LMX1B MNX1 SP8
Fraction of cells in group(%)
20 40 60 80 100
Mean expression in group(%)
0.0
0.5
c
Klein et al.
Ductal
EPs
Fev+
Epsilon
Delta
Alpha
Beta
Hmga1 Smc3 Onecut1 Hmga2 Sox9 Hdac2 Klf5 Sall4 Tcf3 Cdx2 Smad3 Elf3 Hhip Hoxa3 Cdx1 Sox4 Neurog3 Pax4 Pou2f2 Zbtb18 Sp5 Rfx3 Neurod1 Nkx6-1 Fev Ascl2 Klf2 Arx Pax6 Foxo1 Mafb Smad9 Pbx1 Trps1 Foxn2 Isl1 Bach2 Rfx6 Klf12 Jun Egr1 Fos Nkx2-2 Phf21a Gata6 Atf3 Tcf12 Etv1 Elf2 Cux2 Onecut2 Onecut3 Sox5 Klf7 Lmx1b Mnx1
Fraction of cells in group(%)
20 40 60 80 100
Mean expression in group(%)
0
1
2

Figure S9. Dot plots showing the expression of identified TFs regulating SC-EC-enriched genes across pancreatic datasets. Several TFs identified in SC-islet show subtle or no expression in mouse and human pancreatic lineages *in vivo*. Asterisks indicate genes that show very low expression in human or mouse pancreatic cells.

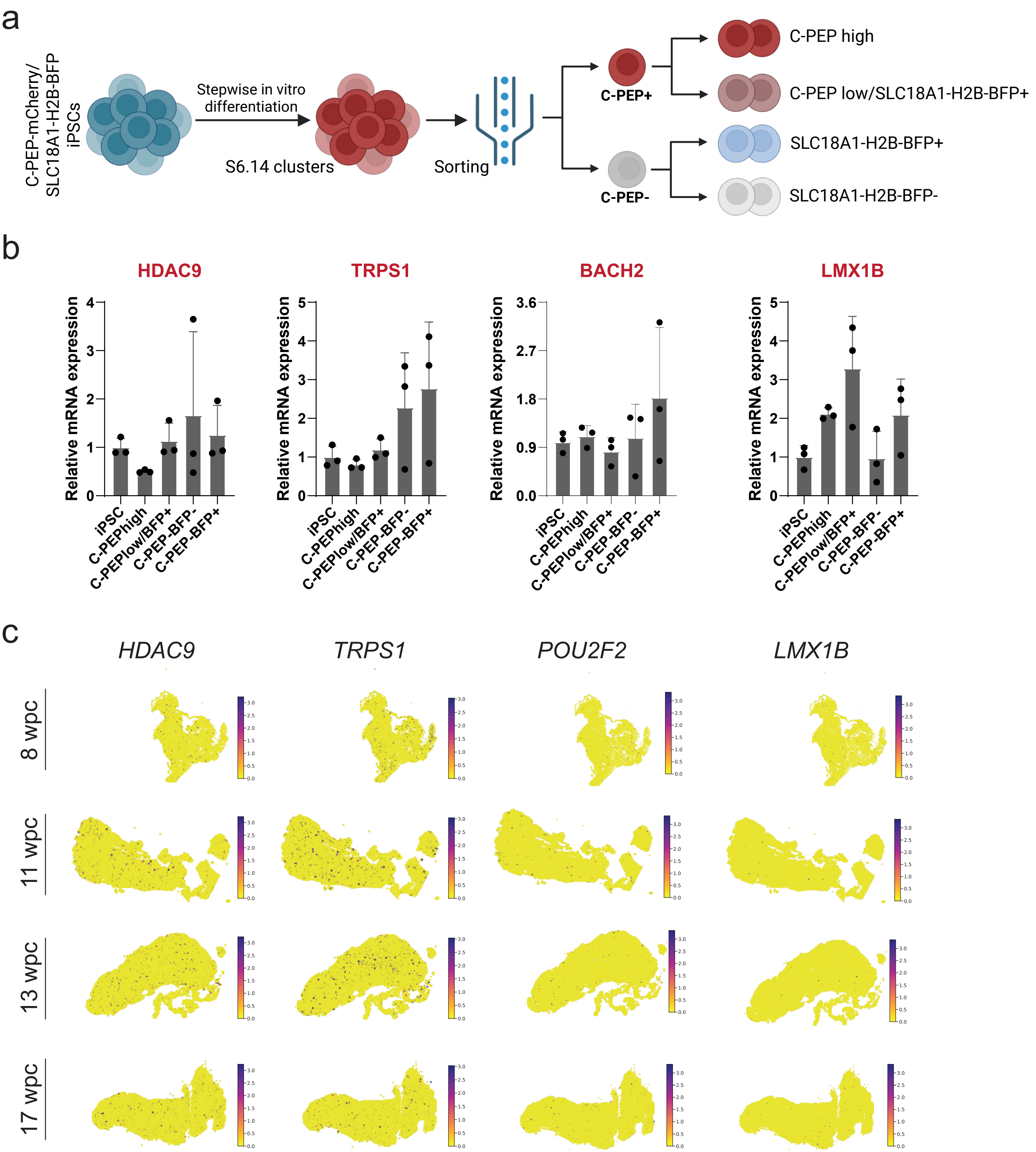

a
C-PEP-mCherry/
SLC18A1-H2B-BFP
iPSCs
Stepwise in vitro
differentiation
S6.14 clusters
Sorting
C-PEP+
C-PEP-
C-PEP high
C-PEP low/SLC18A1-H2B-BFP+
SLC18A1-H2B-BFP+
SLC18A1-H2B-BFP-
b
HDAC9
TRPS1
BACH2
LMX1B
Relative mRNA expression
iPSC
C-PEPhigh
C-PEPlow/BFP+
C-PEP-BFP-
C-PEP-BFP+
c
HDAC9
TRPS1
POU2F2
LMX1B
8 wpc
11 wpc
13 wpc
17 wpc

Figure S10. (a) Schematic of the dual-reporter sorting strategy. C-PEP-mCherry; SLC18A1-H2B-BFP reporter iPSCs were subjected to stepwise *in vitro* differentiation to S6.14 clusters and sorted by C-PEP status. C-PEP$^+$ cells were separated into C-PEP high and C-PEP low (SLC18A1-H2B-BFP$^+$) fractions, and C-PEP$^−$ cells into SLC18A1-H2B-BFP$^+$ and SLC18A1-H2B-BFP$^−$ fractions. (b) qPCR of *HDAC9*, *TRPS1*, *BACH2*, and *LMX1B* across the sorted populations. n = 3; Data are mean ± SD; each dot represents a biological replicate. (c) Spatial expression of *HDAC9*, *TRPS1*, *POU2F2*, and *LMX1B* in the developing human pancreas. *POU2F2* and *LMX1B* are not detected at any stage, while *HDAC9* and *TRPS1* show only low, sparse signal across all stages examined.

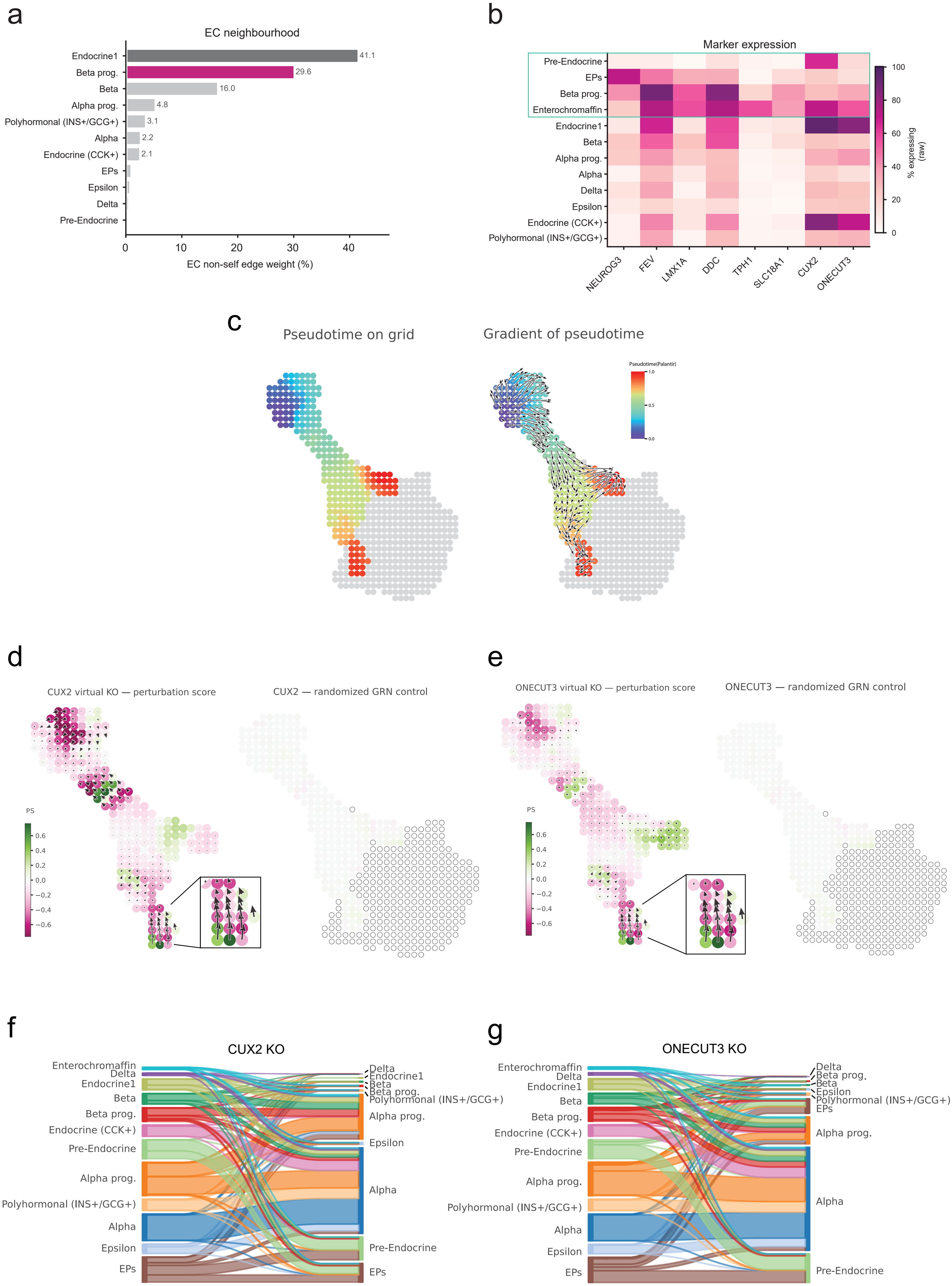
a
EC neighbourhood
Endocrine1
Beta prog.
Beta
Alpha prog.
Polyhormonal (INS+/GCG+)
Alpha
Endocrine (CCK+)
EPs
Epsilon
Delta
Pre-Endocrine
41.1
29.6
16.0
4.8
3.1
2.2
2.1
EC non-self edge weight (%)
b
Marker expression
Pre-Endocrine
EPs
Beta prog.
Enterochromaffin
Endocrine1
Beta
Alpha prog.
Alpha
Delta
Epsilon
Endocrine (CCK+)
Polyhormonal (INS+/GCG+)
NEUROG3
FEV
LMX1A
DDC
TPH1
SLC18A1
CUX2
ONECUT3
% expressing (raw)
c
Pseudotime on grid
Gradient of pseudotime
d
CUX2 virtual KO — perturbation score
CUX2 — randomized GRN control
PS
e
ONECUT3 virtual KO — perturbation score
ONECUT3 — randomized GRN control
f
CUX2 KO
Enterochromaffin
Delta
Endocrine1
Beta
Beta prog.
Endocrine (CCK+)
Pre-Endocrine
Alpha prog.
Polyhormonal (INS+/GCG+)
Alpha
Epsilon
EPs
g
ONECUT3 KO

Figure S11. (a) Test of EC neighbourhood composition across the full dataset. Bars show the percentage of kNN non-self edge weight connecting EC cells to each other population. Endocrine1 (41.1%) and Beta prog. (29.6%) are the largest contributors. Together with panel b, this supports selecting Pre-Endocrine, EPs, Beta prog., and Enterochromaffin as a coherent downstream lineage culminating in the EC fate. (b) Marker gene expression across the neighbourhood populations in panel a. Colour intensity denotes the percentage of cells expressing each gene. EC-specific markers (e.g., *TPH1*, *SLC18A2*) are restricted to Enterochromaffin, while *CUX2*, *ONECUT3*, *FEV*, *DDC*, and *LMX1A* are shared across Enterochromaffin, Pre-Endocrine, and EPs, supporting treatment of these populations as a continuous lineage. (c) Palantir pseudotime on the four-population lineage subset (n = 4288 cells) defined in panels a,b. Left: pseudotime on the analysis grid (40×40; 237 mass-filtered points retained), progressing from early (blue) to late (red). Right: pseudotime gradient vectors, confirming Enterochromaffin as the terminal state. (d, e) In silico knockout of CUX2 and ONECUT3 in the SC-EC lineage. Left, perturbation score (PS; inner product of the simulated shift vector and the reference differentiation field) at each mass-passing grid point, on a green–magenta diverging scale centred at zero (green, positive PS = aligned with differentiation; magenta, negative PS = opposing it); black arrows, simulated shift-vector field; inset, magnified view of the SC-EC region. Right, randomized-GRN control rendered identically. Grid, 40 × 40; n = 237 mass-passing points. (f, g) Markov fate-probability simulations after *CUX2* and *ONECUT3* virtual knockout. Sankey diagrams show predicted fate redistribution.

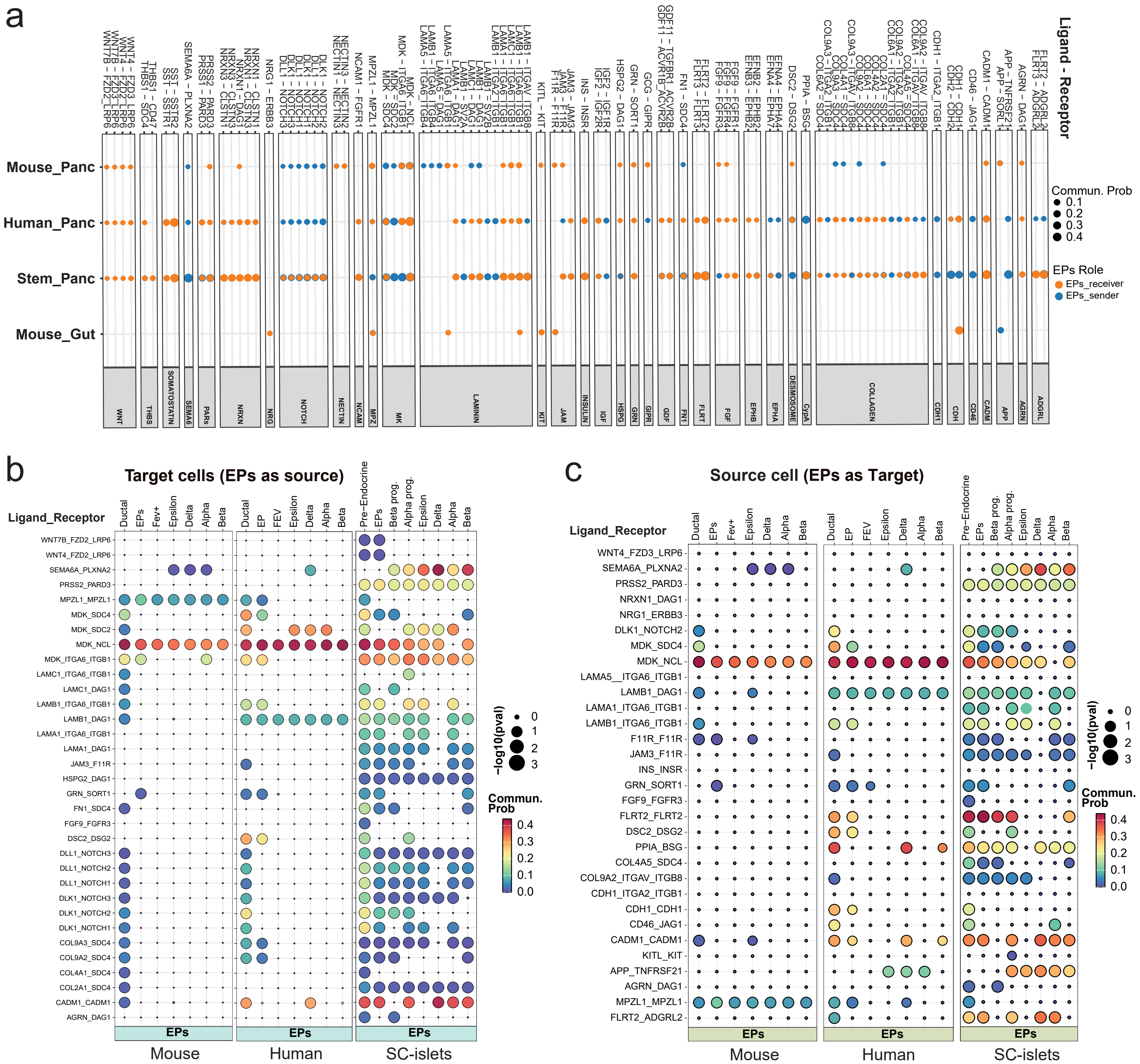
a
Ligand - Receptor
Mouse_Panc
Human_Panc
Stem_Panc
Mouse_Gut
Commun. Prob
0.1
0.2
0.3
0.4
EPs Role
EPs_receiver
EPs_sender
WNT
THBS
SOMATOSTATIN
SEMA6
PARs
NRXN
NRG
NOTCH
NECTIN
NCAM
MPZ
MK
LAMININ
KIT
JAM
INSULIN
IGF
HSPG
GRN
GIPR
GDF
FN1
FLRT
FGF
EPHB
EPHA
DESMOSOME
CypA
COLLAGEN
CDH1
CDH
CD46
CADM
APP
AGRN
ADGRL
b
Target cells (EPs as source)
Ligand_Receptor
Ductal
EPs
Fev+
Epsilon
Delta
Alpha
Beta
EP
FEV
Pre-Endocrine
Beta prog.
Alpha prog.
WNT7B_FZD2_LRP6
WNT4_FZD2_LRP6
SEMA6A_PLXNA2
PRSS2_PARD3
MPZL1_MPZL1
MDK_SDC4
MDK_SDC2
MDK_NCL
MDK_ITGA6_ITGB1
LAMC1_ITGA6_ITGB1
LAMC1_DAG1
LAMB1_ITGA6_ITGB1
LAMB1_DAG1
LAMA1_ITGA6_ITGB1
LAMA1_DAG1
JAM3_F11R
HSPG2_DAG1
GRN_SORT1
FN1_SDC4
FGF9_FGFR3
DSC2_DSG2
DLL1_NOTCH3
DLL1_NOTCH2
DLL1_NOTCH1
DLK1_NOTCH3
DLK1_NOTCH2
DLK1_NOTCH1
COL9A3_SDC4
COL9A2_SDC4
COL4A1_SDC4
COL2A1_SDC4
CADM1_CADM1
AGRN_DAG1
EPs
Mouse
Human
SC-islets
-log10(pval)
0
1
2
3
Commun. Prob
0.4
0.3
0.2
0.1
0.0
c
Source cell (EPs as Target)
Ligand_Receptor
WNT4_FZD3_LRP6
SEMA6A_PLXNA2
PRSS2_PARD3
NRXN1_DAG1
NRG1_ERBB3
DLK1_NOTCH2
MDK_SDC4
MDK_NCL
LAMA5__ITGA6_ITGB1
LAMB1_DAG1
LAMA1_ITGA6_ITGB1
LAMB1_ITGA6_ITGB1
F11R_F11R
JAM3_F11R
INS_INSR
GRN_SORT1
FGF9_FGFR3
FLRT2_FLRT2
DSC2_DSG2
PPIA_BSG
COL4A5_SDC4
COL9A2_ITGAV_ITGB8
CDH1_ITGA2_ITGB1
CDH1_CDH1
CD46_JAG1
CADM1_CADM1
KITL_KIT
APP_TNFRSF21
AGRN_DAG1
MPZL1_MPZL1
FLRT2_ADGRL2
EPs
Mouse
Human
SC-islets
-log10(pval)
0
1
2
3
Commun. Prob
0.4
0.3
0.2
0.1
0.0

Figure S12. (a) Dot plot depicting ligand-receptor (L-R) pairs between different lineages across the four analyzed datasets. The complete list of pairs for each cluster is provided in Supplementary Table 11a-d. (b, c) Dot plots illustrating potential interactions between L-R in pEPs and their corresponding L-R from other pancreatic lineages. Dot size reflects statistical significance (−log10 pvalue), while dot color indicates communication probability, highlighting both conserved and species-specific signaling patterns.

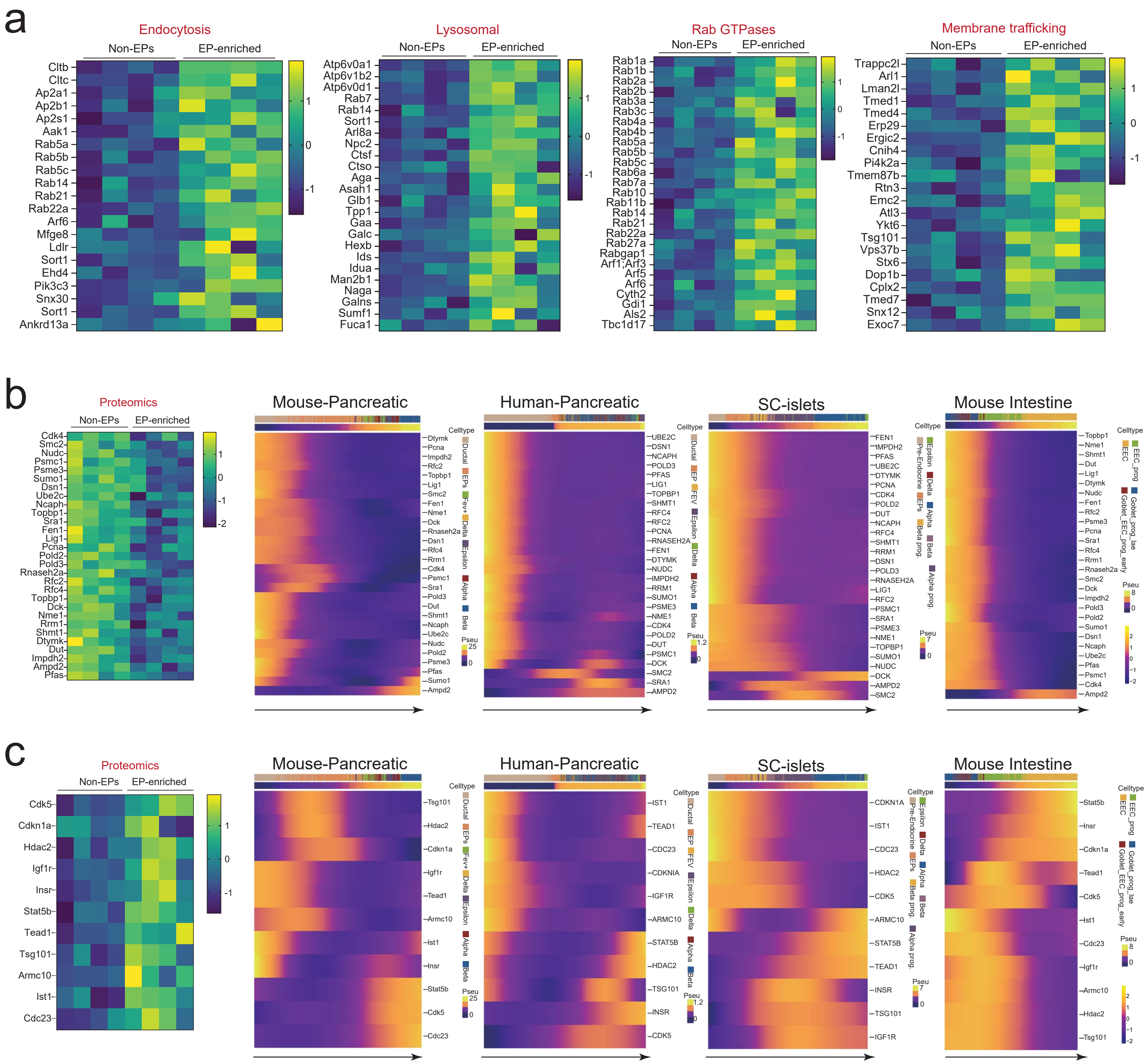

a
Endocytosis
Non-EPs
EP-enriched
Lysosomal
Rab GTPases
Membrane trafficking
b
Proteomics
Mouse-Pancreatic
Human-Pancreatic
SC-islets
Mouse Intestine
Celltype
Pseu
c

Figure S13. (a) Heatmaps showing the expression of multiple proteins involved in the indicated cellular processes in embryonic EP-enriched compared to non-endocrine pancreatic epithelial cells. (b) Heatmaps and pseudotime plots profiling protein and mRNA expression of cell cycle, DNA replication and nucleoside synthesis genes across datasets. (c) Heatmaps and pseudotime plots profiling protein and mRNA expression of cell cycle inhibitors and cell growth genes across datasets. Arrows show directionality.

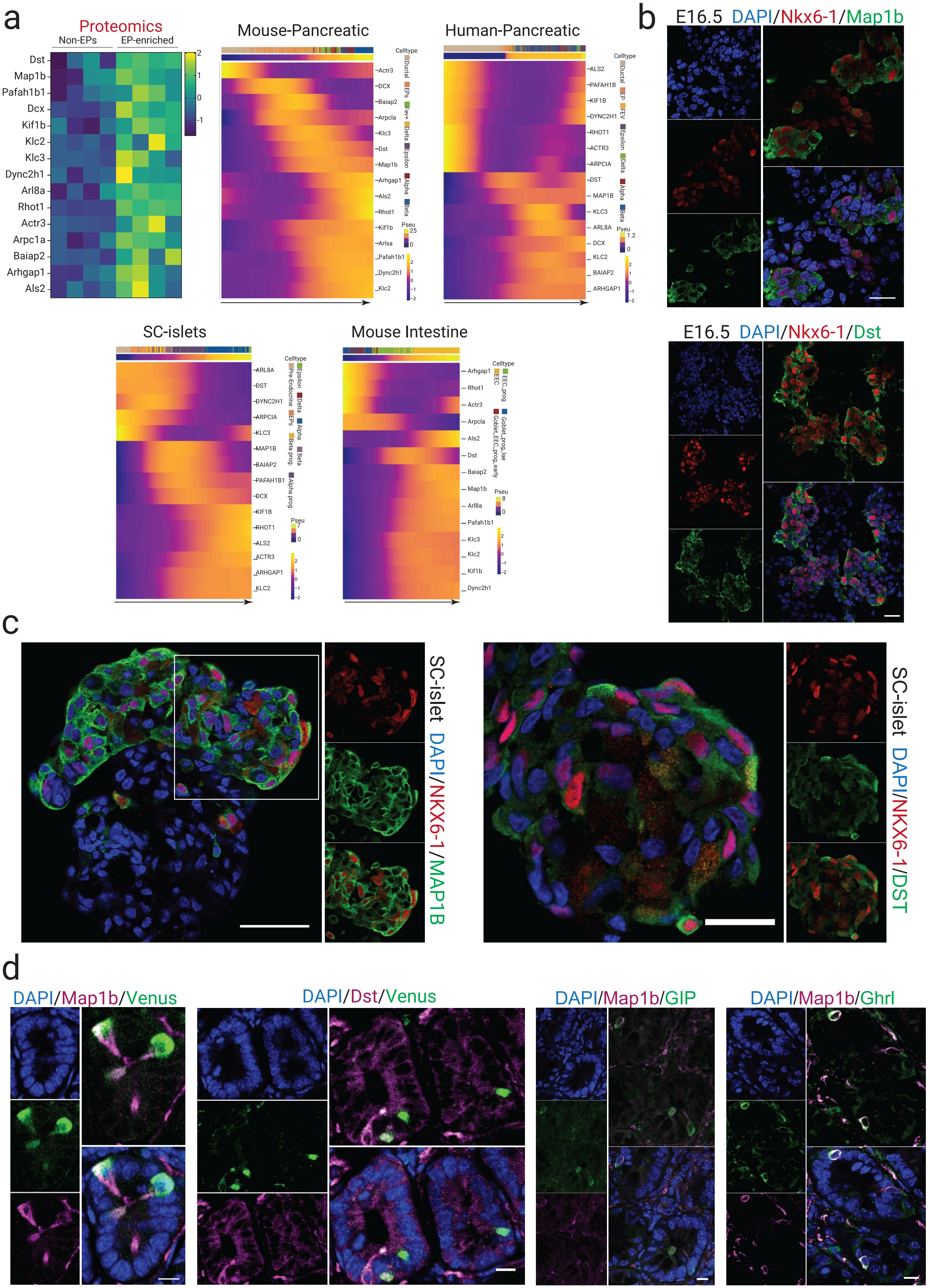
a
Proteomics
Non-EPs
EP-enriched
Dst
Map1b
Pafah1b1
Dcx
Kif1b
Klc2
Klc3
Dync2h1
Arl8a
Rhot1
Actr3
Arpc1a
Baiap2
Arhgap1
Als2
Mouse-Pancreatic
Human-Pancreatic
Celltype
Ductal
EPs
Fev+
Delta
Epsilon
Alpha
Beta
Pseu
SC-islets
Mouse Intestine
b
E16.5 DAPI/Nkx6-1/Map1b
E16.5 DAPI/Nkx6-1/Dst
c
SC-islet DAPI/NKX6-1/MAP1B
SC-islet DAPI/NKX6-1/DST
d
DAPI/Map1b/Venus
DAPI/Dst/Venus
DAPI/Map1b/GIP
DAPI/Map1b/Ghrl

Figure S14. (a) Heatmaps and pseudotime plots profiling protein and mRNA expression of MT-associated genes across datasets. Arrows show directionality. (b-d) Representative confocal images visualizing MAP1B and DST expression in sections from mouse embryonic pancreatic *in vivo* (b), SC-islet differentiation *in vitro* (c), and mouse adult intestinal *in vivo* (d). E refers to embryonic day.

Table S1: List of all identified TFs across different lineages in mouse pancreatic samples and their predicted target genes.

Table S2: (a) List of key TFs in each cell cluster of mouse pancreatic lineages. (b) List of target genes of all cluster-specific TFs for each cluster. (c–f) Pathway enrichment analyses for the target genes of the cluster-specific TFs. (g) TFs regulating genes in the pathways shown in Extended Fig. 2b-e; for each pathway term, the percentage of target genes controlled by specific TFs is calculated.

Table S3: List of all identified TFs across different lineages in human pancreatic samples and their predicted target genes.

Table S4: (a) The number of common target genes for each TF between mouse and human pancreatic lineages. (b) Comparison of NGN3 target genes predicted by $SCENIC^+$ in mouse and human, cross-referenced with NGN3 target genes in mouse pancreas detected by CUT&RUN. (c) Pathway enrichment analyses for the conserved NGN3 target genes from the $SCENIC^+$ analyses of mouse and human and from the CUT&RUN data in mouse pancreas.

Table S5: (a) List of highly expressed genes (HEGs) in pEP/Fev^+ cells in the mouse. (b) Pathway enrichment analyses of HEGs in pEP/Fev^+ cells in the mouse. (c) List of HEGs in pEP/FEV^+ cells in the human. (d) Pathway enrichment analyses of HEGs in pEP/FEV^+ cells in the human. (e) Pathway enrichment analyses of common HEGs in pEP/FEV^+ cells in the mouse and human. (f) List of HEGs in EP/FEV^+ cells in the human pancreas associated with HbA1c/T2D.

Table S6: List of all identified TFs across different lineages in SC-islets and their predicted target genes.

Table S7: (a) The number of common target genes for each TF between *in vivo* human pancreatic lineages and *in vitro* SC-islets. (b) Comparison of NGN3 target genes predicted by $SCENIC^+$ in *in vivo* and *in vitro* human pancreatic cells, cross-referenced with NGN3 target genes in SC-islets detected by CUT&RUN. (c) List of HEGs in pEP/β-progenitors in the SC-islets. (d) Pathway enrichment analyses of HEGs in the human *in vivo* and in the SC-islets. (e) Pathway enrichment analyses of common HEGs in the human *in vivo* and *in vitro* samples.

Table S8: List of all identified TFs across different lineages in mouse intestine and their predicted target genes.

Table S9: (a) List of key TFs in each cell cluster of mouse intestinal lineages. (b) List of common target genes of all key TFs in iEPs and mouse pEPs/Fev$^+$ cells. (c) Pathway enrichment analyses of the common target genes of all key TFs in iEPs and mouse pEPs/Fev$^+$ cells. (d) Comparison of NGN3 target genes predicted by SCENIC$^+$ in mouse and human pancreatic cells and in mouse intestinal cells. (e) Pathway enrichment analyses of NGN3 target genes predicted by SCENIC$^+$ across the three *in vivo* datasets. (f) List of HEGs in iEPs. (g) Pathway enrichment analysis of HEGs in iEPs. (h) List of common HEGs in pEPs and iEPs. (i) Pathway enrichment analysis of common HEGs in pEPs and iEPs. (j) List of HEGs in EP/Fev$^+$ cells in the mouse intestine associated with glycemic risk.

Table S10: (a) List of genes highly expressed in SC-ECs. (b) List of SC-EC-enriched genes that are absent from mouse and human pancreatic lineages *in vivo*. (c) List of predicted TFs regulating the expression of SC-EC-enriched genes. (d) List of predicted TFs regulating the expression of SC-EC-enriched genes in *in vitro* SC-islets and *in vivo* mouse intestinal cells.

Table S11: Ligand–receptor pairs in pEPs and iEPs across all datasets: (a) mouse pEPs; (b) human pEPs; (c) SC-EPs; (d) mouse iEPs.

Table S12: (a) List of all proteins identified in the proteomic analysis of pEP-enriched (pEPe) versus non-pEP cells isolated from the NVF reporter mouse line. (b) List of proteins downregulated in pEP-enriched vs. non-pEP cells. (c) List of proteins upregulated in pEP-enriched vs. non-pEP cells. (d) Pathway enrichment analysis of proteins downregulated in pEP-enriched vs. non-pEP cells. (e) Pathway enrichment analysis of proteins upregulated in pEP-enriched vs. non-pEP cells.
